# Hollow Cu_2_O Nanozymes Enhance Probiotic Therapy for Colitis via Redox Homeostasis and TXNIP/NLRP3 Inflammasome Inhibition

**DOI:** 10.1002/advs.76598

**Published:** 2026-07-14

**Authors:** Guangzhao Wang, Jialing Cao, Pengfei Li, Yujie Wang, Shiqi Lu, Kangliang Sheng, Shan Gao, Yongzhong Wang

**Affiliations:** ^1^ School of Life Sciences and Medical Engineering Anhui University Hefei Anhui China; ^2^ Key Laboratory of Human Microenvironment and Precision Medicine of Anhui Higher Education Institutes Anhui University Hefei Anhui China; ^3^ School of Chemistry and Chemical Engineering Anhui University Hefei Anhui China

**Keywords:** cuprous oxide, gut microbiota, inflammatory bowel disease, nanozymes, reactive oxygen species, TXNIP/NLRP3 inflammasome

## Abstract

Inflammatory bowel disease (IBD) progression is sustained by a positive feedback loop. Excessive mucosal ROS activate the TXNIP/NLRP3 inflammasome axis, which in turn drives interleukin‐1β‐mediated epithelial barrier disruption and dysbiosis. Conventional solid nanozymes suffer from limited catalytic efficiency because dense interiors restrict substrate access. Here, hollow cuprous oxide nanozymes (H‐Cu_2_O) are engineered to overcome these limitations. The hollow architecture exposes a larger catalytically accessible surface area, enabling H‐Cu_2_O to achieve broad‐spectrum reactive oxygen species scavenging with superior efficiency compared with its composition‐matched solid counterpart (S‐Cu_2_O). In mouse models of colitis, low‐dose H‐Cu_2_O (4 mg kg^−1^) attenuates oxidative damage, suppresses the TXNIP/NLRP3 cascade, and restores tight junction integrity. Furthermore, 16S rRNA sequencing reveals that H‐Cu_2_O remodels the dysbiotic gut microbiota toward homeostasis with Lactobacillus enrichment. Importantly, the sequential co‐administration of Lactiplantibacillus plantarum with H‐Cu_2_O outperforms the electrostatically assembled hybrid LP@H‐Cu_2_O, demonstrating that free nanozyme diffusion is more effective than surface immobilization for combination therapy. This work establishes hollow nanozyme architecture as a key determinant of anti‐inflammatory efficacy and validates nanozyme‐probiotic co‐delivery as a translatable IBD treatment strategy.

## Graphical abstract

Hollow cuprous oxide (H‐Cu_2_O) nanozymes feature enlarged catalytic surfaces for superior reactive oxygen species (ROS) scavenging. By efficiently neutralizing mucosal ROS, H‐Cu_2_O directly suppresses the TXNIP/NLRP3 inflammasome axis and restores intestinal epithelial barrier integrity. This structural engineering overcomes diffusion limitations of solid nanozymes, establishing a highly efficient nanotherapeutic strategy for treating inflammatory bowel disease.

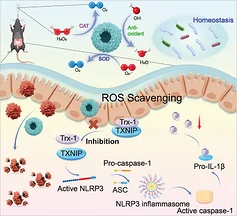

## 1 Introduction

Inflammatory bowel disease (IBD) afflicts millions worldwide with chronic, relapsing intestinal inflammation that remains inadequately controlled by existing therapies [1, 2, 3]. A primary driver of IBD pathogenesis is the self‐amplifying cycle between excessive mucosal reactive oxygen species (ROS) and inflammation activation [4, 5, 6]. Therefore, breaking this loop of ROS, inflammation, and intestinal barrier destruction represents a compelling therapeutic strategy [7, 8, 9, 10]. However, clinical translation of conventional antioxidant therapies faces many difficulties [11, 12, 13]. The small‐molecule antioxidants are limited by poor target selectivity and rapid systemic metabolic clearance [14, 15, 16, 17]. Moreover, recombinant antioxidant enzymes encounter further limitations in vivo, including proteolytic degradation, immunogenicity, and pH‐induced denaturation within the gastrointestinal tract, all of which undermine their functional efficacy [18, 19]. To address these limitations, metal‐based nanozymes have emerged as alternatives with sustained enzyme‐mimetic catalytic performance [20, 21, 22]. In particular, copper‐based nanozymes are a uniquely promising therapeutic platform. Their reversible Cu^+^/Cu^2+^ redox couple can simultaneously activate multiple antioxidant pathways. Meanwhile, they have the key advantages of simple structure, low cost, and single‐component design [23, 24]. These distinct benefits have driven rapidly growing interest in their therapeutic research and development.

Despite these advances, three interrelated challenges hinder the clinical translation of nanozyme‐based IBD therapy. First, solid cuprous oxide nanozymes show suboptimal catalytic efficiency at biosafety doses because most catalytic centers are buried and inaccessible to substrates [25, 26, 27, 28]. Second, the mechanistic link between ROS scavenging and inflammasome regulation remains poorly understood [29]. Third, while nanomaterial‐bacteria hybrid combination has been considered for IBD therapy, most methods focus on physical immobilization or encapsulating nanomaterials on probiotic surfaces [30]. These approaches are complex, may damage bacterial membranes, and limit the nanozyme's diffusion to nearby areas, reducing its ability to effectively scavenge ROS across a wider gut microenvironment necessary for mucosal redox balance [31, 32, 33, 34].

Hollow nanostructure engineering provides a strategy for catalytic performance optimization [35, 36]. Replacing the dense solid core with an interior cavity fully exposes both inner and outer surfaces for catalytic reactions, drastically shortens mass diffusion pathways, and increases the density of accessible active sites, all without altering the material's elemental composition [37, 38]. Meanwhile, a unique co‐administration method that maintains the independent functions of nanozymes and probiotics while allowing their coordinated action in vivo has been largely unexplored [39, 40].

Here, we designed hollow cuprous oxide nanozymes (H‐Cu_2_O) that exhibit superior ROS scavenging abilities compared to solid counterparts (S‐Cu_2_O) due to their unique structure. In a mouse model of colitis, a low dose of H‐Cu_2_O (4 mg kg^−1^) effectively maintains the reduced thioredoxin‐1 (Trx‐1)/thioredoxin‐interacting protein (TXNIP) inhibitory complex, preventing TXNIP‐related NLRP3 inflammasome assembly and IL‐1β maturation. Furthermore, 16S rRNA sequencing reveals that H‐Cu_2_O‐mediated redox homeostasis drives selective enrichment of Lactobacillus via alleviating oxidative selective pressure on microaerophilic commensal bacteria (Scheme 1). Based on these findings, we propose a functionally independent and spatiotemporally coordinated methodology by a sequential co‐administration combining H‐Cu_2_O with *Lactiplantibacillus plantarum* (LP), without employing a hybrid combination treatment. This mixture surpasses the electrostatic adsorption hybrid bacteria nanozyme combination treatment (LP@H‐Cu_2_O) in protecting the microbiota and ameliorating colitis in mice. Together, this work identifies hollow nanozyme architecture as a critical determinant of therapeutic efficacy at the inflammasome signaling level, and validates the nanozyme probiotic co‐delivery strategy as a translationally promising method for IBD nanomedicine.

**SCHEME 1:**
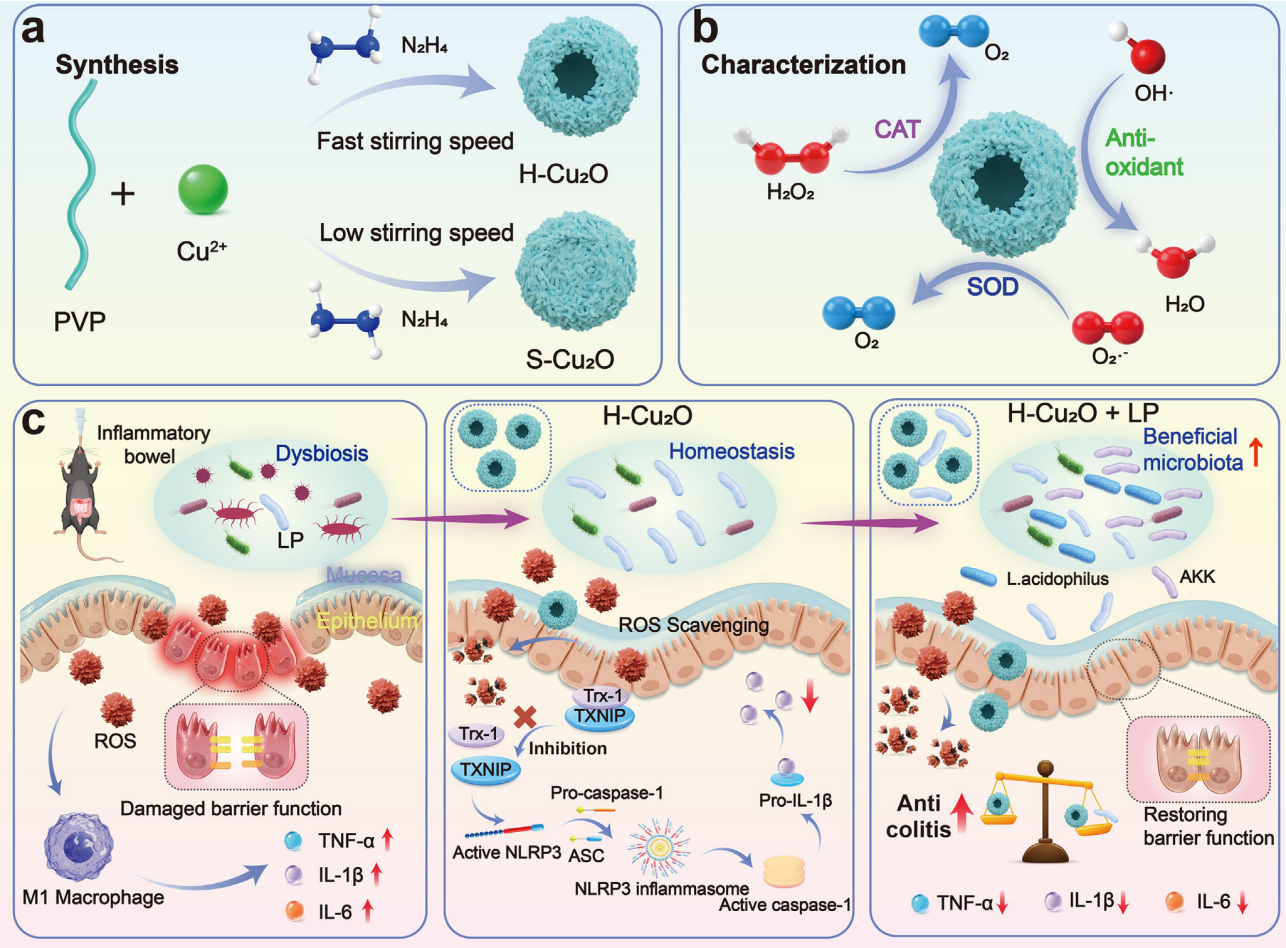
Design, ROS‐scavenging mechanism, and in vivo therapeutic strategy of hollow Cu_2_O nanozymes. (a) Schematic illustration of H‐Cu_2_O and S‐Cu_2_O synthesis. (b) CAT‐/SOD‐mimicking and antioxidant activities of Cu_2_O nanozymes. (c) H‐Cu_2_O scavenges mucosal ROS, suppresses the TXNIP/NLRP3 inflammasome cascade, and synergizes with *L. plantarum* (LP) co‐administration to restore gut homeostasis.

## 2 Results and Discussion

### 2.1 Synthesis and Characterization of H‐Cu_2_O and S‐Cu_2_O Nanozymes

To investigate the effect of the morphology on the enzyme activity, cuprous oxide nanozymes with different morphologies were synthesized using a surfactant confinement strategy. By finely tuning the surfactant addition amounts of the poly(vinylpyrrolidone) (PVP), stirring rates, and hydrothermal durations, the crystal growth process of cuprous oxide nanozymes was selectively directed toward either dense particle formation or internal cavitation [41]. As shown in Figure 1a,b, the morphologies of the obtained samples were hollow and solid nanospheres, respectively. Energy‐dispersive X‐ray spectroscopy (EDS) mapping and X‐ray photoelectron spectroscopy (XPS) consistently demonstrated a uniform spatial distribution of Cu and O, with Cu LMM Auger spectra further corroborating a predominantly Cu^+^/O^2−^ inorganic core (Figure 1c,g–i). X‐ray diffraction (XRD) analysis confirmed that both architectures crystallize into the standard phase‐pure Cu_2_O framework (PDF#74‐1230) without extraneous inorganic impurities (Figure 1d) [42]. Notably, the intended uniform surface modification was verified: both the XPS survey and EDS detected distinct N signals accompanying the structural elements, while Fourier‐transform infrared (FTIR) spectroscopy revealed characteristic C─N and C═O stretching vibrations (Figure 1c,e,f). Based on these characterizations, the solid and hollow architectures are prepared (named S‐Cu_2_O and H‐Cu_2_O), respectively, providing a well‐defined materials model for subsequent experiments.

**FIGURE 1:**
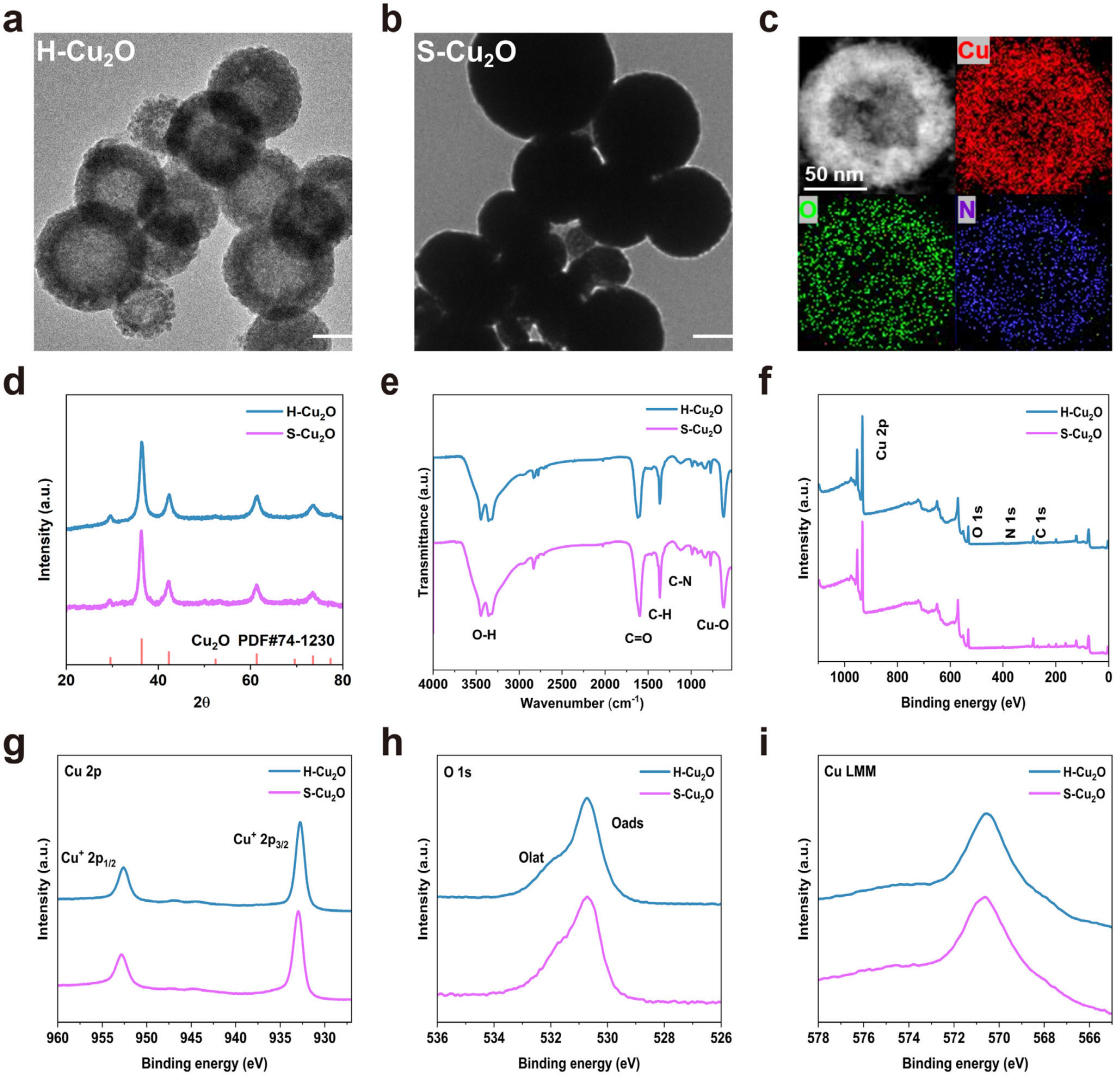
Characterization of H‐Cu_2_O and S‐Cu_2_O nanozymes. (a, b) TEM images of H‐Cu_2_O and S‐Cu_2_O (scale bar: 50 nm). (c) EDS elemental mapping images showing the spatial distribution of (Cu, O, and N, scale bar: 50 nm). (d) XRD patterns and (e) FTIR spectra of H‐Cu_2_O and S‐Cu_2_O, respectively. (f) XPS survey spectra, (g) high‐resolution Cu 2p, (h) High‐resolution O 1s XPS spectra, and (i) Cu LMM Auger spectra of H‐Cu_2_O and S‐Cu_2_O.

To explore the structural information of the two samples, we performed DLS size analysis, zeta potential measurements, and BET/BJH surface characterization for both Cu_2_O nanozymes (Figures S1–S3). DLS revealed that both H‐Cu_2_O and S‐Cu_2_O display narrow size distributions with principal peaks centred at approximately 250 nm (Figure S1), confirming size uniformity. Zeta potential measurements gave values of 3.054 ± 0.207 mV for H‐Cu_2_O and 2.877 ± 0.328 mV for S‐Cu_2_O (Figure S2), indicating similar surface charge and colloidal stability, thus excluding electrostatic effects as a cause for activity differences. Furthermore, BET analysis demonstrated that the specific surface area and the mesopore volume of H‐Cu_2_O were higher than those of S‐Cu_2_O (Figure S3). Therefore, the special hollow structure of the Cu_2_O would expose more catalytic surfaces and reduce diffusion resistance, enhancing active‐site density, and substrate transport. These structural benefits support H‐Cu_2_O's superior ROS‐scavenging and enzyme‐mimicking abilities.

### 2.2 Hollow Architecture Confers Superior Enzyme‐Mimicking Kinetics and Broad‐Spectrum ROS Scavenging

Having confirmed their structural and compositional equivalence, we systematically compared the enzyme‐mimicking and broad‐spectrum ROS‐scavenging capacities of H‐Cu_2_O and S‐Cu_2_O. CAT‐like activity was evaluated by monitoring H_2_O_2_ decomposition and dissolved oxygen generation, whereas SOD‐like activity and radical‐scavenging capacity were assessed using O_2_•^−^, OH•, DPPH•, and ABTS•+ assays. Michaelis‐Menten kinetic analysis was further performed using H_2_O_2_ as the substrate. Mechanistically, the Cu_2_O nanozymes utilize the Cu^+^/Cu^2+^ redox cycle and interfacial electron transfer to simultaneously drive catalase (CAT)‐ and superoxide dismutase (SOD)‐like pathways (Figure 2a). Specifically, the CAT‐mimicking activity catalyzes H_2_O_2_ decomposition into O_2_, thereby preventing H_2_O_2_‐driven Fenton‐type cascades. Concurrently, the SOD‐mimicking activity accelerates the disproportionation of O_2_•^−^, mitigating downstream OH• generation. Furthermore, H‐Cu_2_O effectively scavenges organic radical models (DPPH• and ABTS•+), confirming its broad‐spectrum antioxidant profile.

**FIGURE 2:**
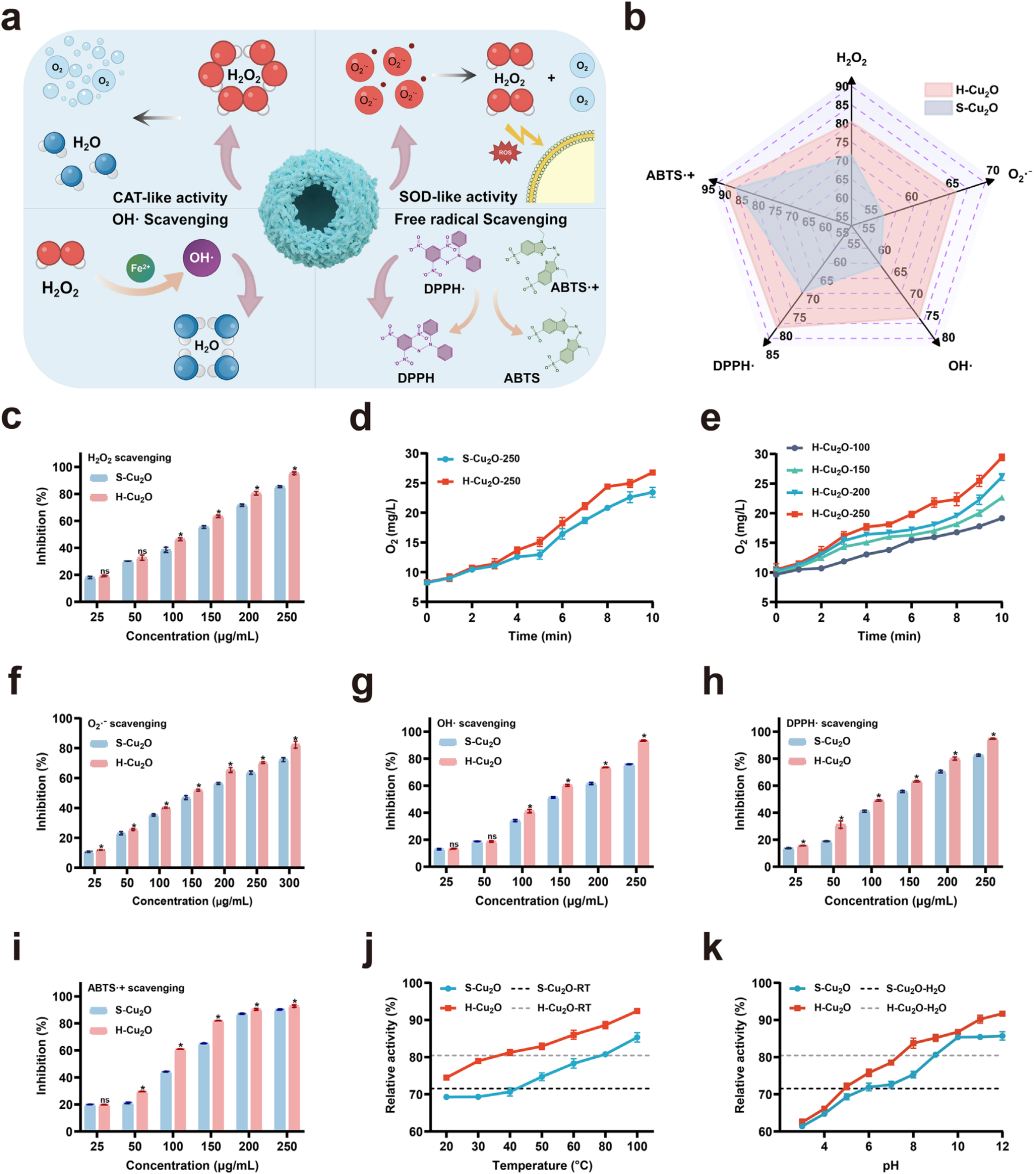
Enzyme‐mimicking activities and broad‐spectrum ROS‐scavenging capacities of Cu_2_O nanozymes. (a) Schematic illustration of Cu_2_O nanozyme‐mediated CAT‐ and SOD‐mimicking pathways. (b) Radar plot summarizing the integrated ROS‐scavenging performance of H‐Cu_2_O and S‐Cu_2_O. (c, f–i) Concentration‐dependent scavenging efficiencies toward H_2_O_2_, O_2_•^−^, OH•, DPPH•, and ABTS•+. (d) Dissolved oxygen (DO) generation kinetics at a fixed dose of 250 µg mL^−1^. (e) Concentration‐dependent DO generation kinetics for H‐Cu_2_O. (j, k) H_2_O_2_ scavenging efficiencies of H‐Cu_2_O across varied temperatures (j) and physiological or pathological pH ranges (k). Data are presented as mean ± SD. ^*^ *p* < 0.05, ^**^ *p* < 0.01, ^***^ *p* < 0.001.

A comprehensive multi‐dimensional analysis visually demonstrates that H‑Cu_2_O exhibits markedly superior ROS scavenging performance relative to S‑Cu_2_O across all five evaluated parameters (Figure 2b). Concentration‐dependent assays (25–250 µg mL^−1^) reveal that H‐Cu_2_O maintains a significantly higher H_2_O_2_ elimination efficiency across the entire kinetic range (Figure 2c). Dissolved oxygen (DO) monitoring further validates this kinetic advantage at a fixed dose (250 µg mL^−1^). H‐Cu_2_O triggers a more rapid initial O_2_ evolution and achieves a higher steady‐state yield than S‐Cu_2_O (Figure 2d), with catalytic outputs scaling proportionally with the administered dose (Figure 2e). Importantly, H‐Cu_2_O also consistently outperforms S‐Cu_2_O in neutralizing O_2_•^−^, OH•, and organic radicals (Figure 2f–i). This enhanced catalytic performance is achieved through morphological engineering alone. Because elemental composition is held constant by the S‐Cu_2_O control, the activity improvement is attributable solely to the hollow architecture, which confers three advantages: exposure of internal surfaces, increasing active‐site density; shorter intra‐particle diffusion paths; and expanded interfacial area for accelerated electron transfer.

To rigorously substantiate these kinetic claims, we performed Michaelis‐Menten kinetic analysis for the CAT‐like activity of both nanozymes using H_2_O_2_ as the varying substrate at a fixed nanozyme concentration (Figure S4). Michaelis‐Menten fitting and Lineweaver‐Burk double‐reciprocal plots consistently show that H‐Cu_2_O exhibits a lower apparent Km and a higher Vmax than S‐Cu_2_O. The lower Km of H‐Cu_2_O reflects higher substrate affinity enabled by the greater density of accessible active sites on the hollow interior surface (BET: 18.93 vs. 10.32 m^2^/g), while the higher Vmax reflects faster maximum catalytic throughput facilitated by the shortened intra‐particle diffusion pathways. The apparent catalytic efficiency, expressed as Vmax/Km, is correspondingly higher for H‐Cu_2_O than for S‐Cu_2_O, providing direct quantitative evidence that the hollow architecture confers superior CAT‐like kinetics. For SOD‐like activity, H‐Cu_2_O achieves significantly higher enzymatic activity than S‐Cu_2_O across the full tested concentration range (Figure S5), consistent with the superior O_2_•^−^ scavenging efficiency observed in Figure 2f and attributable to the same morphology‐driven increase in accessible active‐site density.

This structure‐activity relationship is directly corroborated by the BET and BJH characterization data (Figure S3). H‐Cu_2_O exhibits a specific surface area of 18.93 m^2^/g, approximately 1.8‐fold higher than S‐Cu_2_O (10.32 m^2^/g), and a substantially larger mesopore volume, demonstrating that the hollow architecture quantitatively increases the accessible catalytic surface area and reduces intra‐particle diffusion resistance. On a physicochemical baseline where particle size (250 nm; Figure S1) and surface charge (zeta potential 3 mV; Figure S2) are matched between H‐Cu_2_O and S‐Cu_2_O, this morphology‐driven surface area enhancement provides direct quantitative support for the superior enzyme‐mimicking kinetics and ROS‐scavenging efficiency of H‐Cu_2_O.

This finding aligns with recent reports emphasizing that mass‐transport limitations, rather than intrinsic site reactivity, often constitute the primary bottleneck for nanozyme therapeutic performance [43, 44, 45]. Compared to conventional single pathway oxides (CeO_2_ and Mn_3_O_4_) or complex noble metal systems (Pt and Au), H‐Cu_2_O uniquely integrates robust CAT/SOD‐mimicking activities within a cost‐effective, single‐component platform [46, 47, 48, 49]. Additionally, H‐Cu_2_O demonstrates exceptional functional stability, retaining robust H_2_O_2_ scavenging activity across a broad thermal range (20–100°C; Figure 2j) and diverse pH conditions (pH 4–12; Figure 2k). This environmental adaptability is particularly valuable under the acidic conditions of the inflamed gastrointestinal tract. Natural antioxidant enzymes typically denature and lose function in such microenvironments. H‐Cu_2_O circumvents this critical vulnerability [50].

### 2.3 H‐Cu_2_O Nanozymes Exert Cytoprotective, Anti‐Inflammatory, and Barrier‐Restorative Effects In Vitro

Building on these abiotic ROS‐scavenging results, we evaluated the cytoprotective, anti‐inflammatory, and barrier‐restorative effects of H‐Cu_2_O using H_2_O_2_‐challenged NCM‐460 colonic epithelial cells and LPS‐stimulated RAW264.7 macrophages. Cell viability and intracellular ROS levels were assessed by CCK‐8 and DCFH‐DA fluorescence assays, respectively. Inflammatory cytokines and epithelial tight‐junction markers were quantified by qPCR and ELISA, while cellular uptake of RhB‐labeled H‐Cu_2_O was examined by confocal laser scanning microscopy (Figure 3a). CCK‐8 assays confirmed the excellent biocompatibility of the nanozymes, with NCM‐460 cell viability maintained above 90% following 24 h incubation at concentrations up to 62.5 µg mL^−1^ (Figure 3c). Therefore, 25 and 50 µg mL^−1^ were chosen as optimal working concentrations for subsequent experiments. Under H_2_O_2_‑induced oxidative stress, H‑Cu_2_O treatment significantly mitigated the dose‑dependent decline in NCM‑460 cell viability, confirming its potent intracellular cytoprotective activity (Figure 3d). To clarify whether the observed intracellular ROS reduction arises from cellular internalization of H‐Cu_2_O or from extracellular scavenging alone, we performed dedicated cellular uptake studies using Rhodamine B‐labeled H‐Cu_2_O (RhB‐H‐Cu_2_O) and confocal laser scanning microscopy. Concentration‐dependent imaging (Figure S6) revealed clear, dose‐dependent intracellular accumulation of RhB fluorescence within the NCM‐460 cytoplasm, well‐separated from the plasma membrane (DiO, green) and the nucleus (DAPI, blue), confirming endocytic internalization rather than surface adsorption. Time‐dependent imaging at 50 µg mL^−1^ (Figure S7) demonstrated progressive cytoplasmic accumulation beginning at 2 h, with a punctate vesicle‐like distribution pattern consistent with endolysosomal trafficking. These findings establish that H‐Cu_2_O is actively internalized by NCM‐460 cells in a time‐ and concentration‐dependent manner. Accordingly, the intracellular ROS clearance visualized by DCFH‐DA (Figure 3b and Figure S8) reflects the combinatorial contributions of extracellular H_2_O_2_ scavenging by nanozymes present in the culture medium and direct intracellular catalytic activity by internalized H‐Cu_2_O within the cytoplasmic compartment.

**FIGURE 3:**
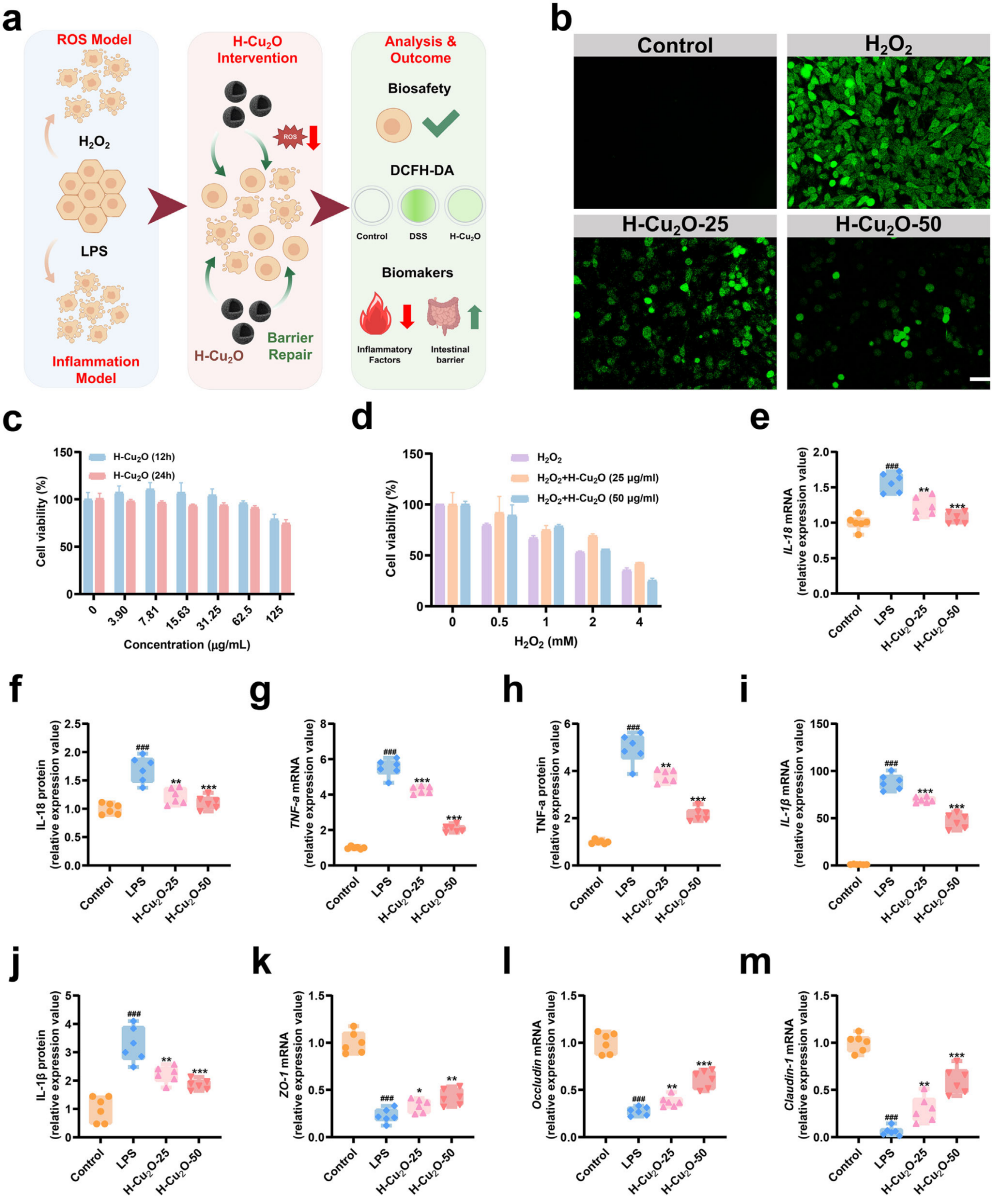
In vitro biological evaluation of H‐Cu_2_O nanozymes. (a) Schematic of the in vitro workflow assessing biosafety, intracellular ROS clearance, immunomodulation, and barrier protection under oxidative and inflammatory stress. (b) Intracellular ROS levels in H_2_O_2_‐challenged NCM‐460 cells following H‐Cu_2_O intervention (DCFH‐DA fluorescence imaging; scale bar: 50 µm). (c) NCM‐460 cell viability after 12 and 24 h exposure to varying concentrations of H‐Cu_2_O (CCK‐8 assay). (d) Cytoprotective effects of H‐Cu_2_O (25 and 50 µg mL^−1^) on NCM‐460 cells under escalating H_2_O_2_ challenges. (e, g, i) Relative mRNA expression levels of *IL‐18*, *TNF‐α*, and *IL‐1β* in LPS‐stimulated RAW264.7 macrophages. (f, h, j) Secreted protein levels of IL‐18, TNF‐α, and IL‐1β in the culture medium of LPS‐stimulated RAW264.7 cells (ELISA). (k–m) Relative mRNA expression levels of tight junction genes (*ZO‐1*, *Occludin*, and *Claudin‐1*) in LPS‐stimulated NCM‐460 cells. Data are presented as mean ± SD. ^#^ *p* < 0.05, ^##^ *p* < 0.01, ^###^ *p* < 0.001 (vs. Control); ^*^ *p* < 0.05, ^**^ *p* < 0.01, ^***^ *p* < 0.001 (vs. LPS or H_2_O_2_ induced model).

To elucidate the downstream anti‐inflammatory cascades, lipopolysaccharide (LPS)‐stimulated RAW264.7 macrophages were utilized as a model of inflammation hyperactivation. H‐Cu_2_O treatment substantially suppressed the LPS‐induced upregulation of pro‐inflammatory cytokines, specifically *IL‐18*, *TNF‐α*, and *IL‐1β* mRNA levels (Figure 3e,g,i). Corresponding enzyme‐linked immunosorbent assay (ELISA) analyses confirmed a parallel dose‐dependent reduction in secreted cytokine proteins (Figure 3f,h,j), demonstrating comprehensive blockade of the inflammatory response at both transcriptional and protein levels.

Given that persistent oxidative stress and inflammatory activation are hallmark drivers of epithelial barrier dysfunction, we further quantified tight junction gene expression in LPS‐induced NCM‐460 cells. LPS‐induced downregulation of key barrier related mRNA levels (*ZO‐1*, *Occludin*, and *Claudin‐1*) was obviously restored by H‐Cu_2_O administration in a dose‐dependent manner (Figure 3k–m). Taken together, these dual‐cell in vitro analyses confirm that H‐Cu_2_O simultaneously suppresses intracellular oxidative stress, restrains macrophage‐mediated inflammatory cascades, and enhances epithelial tight junction integrity, establishing a cellular‐level basis for mucosal homeostasis restoration [51, 52, 53]. A limitation of the present study concerns the use of NCM‐460 cells as the in vitro model of normal colonic epithelium. Future work employing intestinal organoids will provide more physiologically relevant validation of the barrier protective effects reported here.

### 2.4 H‐Cu_2_O Nanozymes Ameliorate DSS‐Induced Murine Colitis In Vivo

Following the in vitro validation, the therapeutic efficacy of H‐Cu_2_O was evaluated in a DSS‐induced acute colitis model in male C57BL/6J mice (Figure 4a). Colitis was induced by administering 2.5% DSS in drinking water, followed by oral treatment with H‐Cu_2_O at 4 mg kg^−1^. Therapeutic outcomes were evaluated by monitoring body weight, disease activity index (DAI), colon length, histopathological changes, oxidative‐stress biomarkers, and epithelial barrier‐related gene and protein expression [54]. DSS administration elicited severe colitic phenotypes characterized by progressive weight loss, an elevated disease activity index, and significant colon shortening. In contrast, oral H‐Cu_2_O intervention markedly mitigated these macroscopic pathological parameters. It significantly attenuated weight loss (Figure 4b), reduced DAI scores (Figure S9), and restored colon length (Figure 4c,d and Figure S10).

**FIGURE 4:**
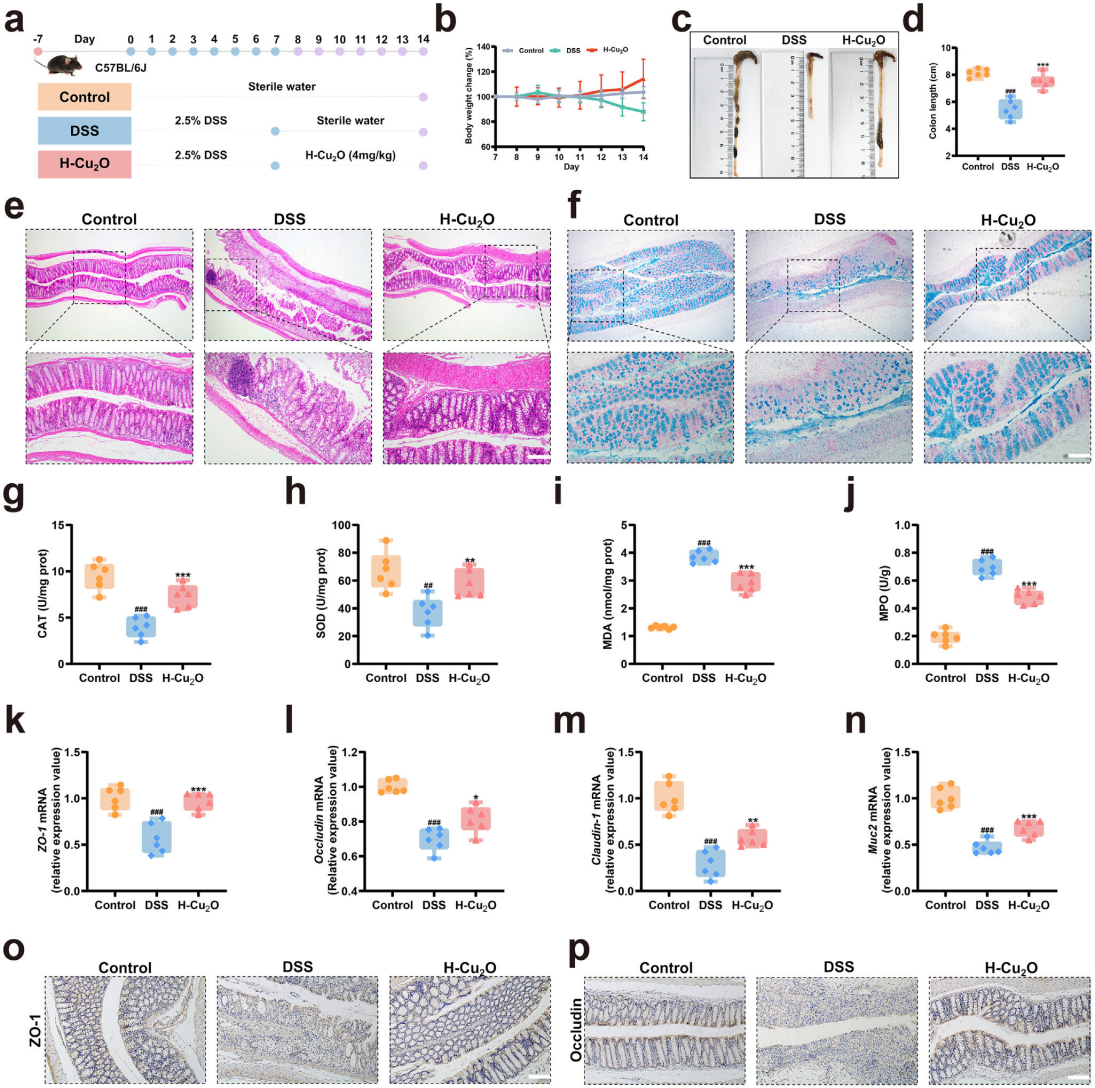
In vivo therapeutic efficacy of H‐Cu_2_O nanozymes in DSS‐induced murine colitis. (a) Schematic representation of the DSS‐induced colitis model and the H‐Cu_2_O treatment protocol. (b) Percentage change in body weight throughout the treatment period. (c) Representative macroscopic images of colons. (d) Quantitative analysis of colon lengths. (e, f) Representative H&E (e) and PAS (f) stained colon sections, with magnified views provided below (scale bars: 50 µm). (g–j) Colonic enzymatic activities and oxidative biomarkers: CAT activity (g), SOD activity (h), MDA content (i), and MPO activity (j). (k–n) Relative mRNA expression levels of colonic tight junction and mucin genes (*ZO‐1*, *Occludin*, *Claudin‐1*, and *Muc2*). (o, p) Immunohistochemical staining for ZO‐1 (o) and Occludin (p) in colon tissues (scale bars: 50 µm). Data are presented as mean ± SD. ^#^ *p* < 0.05, ^##^ *p* < 0.01, ^###^ *p* < 0.001 (*vs*. Control); ^*^ *p* < 0.05, ^**^ *p* < 0.01, ^***^ *p* < 0.001 (vs. DSS).

Histopathological evaluations confirmed the comprehensive intestinal mucosal protective efficacy of H‐Cu_2_O in the DSS‐induced colitis model. Hematoxylin and eosin (H&E) staining of colon sections from DSS‐challenged mice exhibited severe mucosal architectural distortion, complete crypt ablation, and extensive inflammatory cell infiltration (Figure 4e), which aligns with the drastically reduced histological scores (Figure S11). Furthermore, periodic acid‐Schiff (PAS) staining demonstrated that H‐Cu_2_O treatment markedly reversed the DSS‐induced goblet cell depletion and mucus layer thinning, restoring goblet cell populations to near‐homeostatic levels (Figure 4f and Figure S12). These histological data together indicate that H‐Cu_2_O actively promotes mucosal structural repair and exerts potent anti‐inflammatory effects.

To explore the mechanism underlying the therapeutic benefits of H‐Cu_2_O via modulation of colonic redox homeostasis, we quantified the activities of core endogenous antioxidant enzymes and oxidative damage biomarkers in colon tissues. DSS challenge markedly depleted colonic CAT and SOD activities and elevated MDA content and MPO activity, indicating breakdown of antioxidant defenses and intense oxidative stress [55, 56, 57]. H‐Cu_2_O restored CAT and SOD activities and reduced MDA and MPO levels (Figure 4g–j). These findings provide direct evidence that H‐Cu_2_O scavenges ROS in vivo, restores redox homeostasis, and exerts therapeutic benefit [58].

H‐Cu_2_O reversed the DSS‐induced transcriptional downregulation of critical tight junction and mucin genes (*ZO‐1*, *Occludin*, *Claudin‐1*, and *Muc2*) (Figure 4k–n). This transcriptional recovery was confirmed at the protein level by immunohistochemistry, which revealed significant restoration of ZO‐1 and Occludin expression at the epithelial barrier (Figure 4o,p and Figures S13 and S14). Together, these findings indicate that H‐Cu_2_O‐mediated redox regulation directly alleviates colonic inflammation, thereby preserving epithelial tight junctions and the mucus barrier.

Additionally, the biosafety of H‐Cu_2_O was systematically assessed. Histological examination of liver tissues revealed no apparent pathological alterations (Figure S15). In parallel, serum biochemical markers of hepatic function (ALT, AST) and GSH levels were comparable to those of healthy controls (Figure S15). Overall, these results confirm the favorable biosafety profile of H‐Cu_2_O at the therapeutically effective dose.

### 2.5 H‐Cu_2_O inhibits Colonic Inflammation Associated With Suppression of the TXNIP/NLRP3 Inflammasome Axis

Having established that H‐Cu_2_O effectively alleviates colonic oxidative stress and macroscopic inflammation in vivo, we next explored the underlying molecular mechanism, focusing on the redox‐sensitive TXNIP/NLRP3 inflammasome axis. This pathway acts as a critical hub transducing ROS accumulation into caspase‐1‐dependent IL‐1β maturation. Under physiological conditions, reduced thioredoxin‐1 (Trx‐1) binds TXNIP and holds it in an inactive complex. Under oxidative stress, Trx‐1 becomes oxidized and releases TXNIP. The freed TXNIP then associates with NLRP3 and triggers inflammasome assembly [59, 60]. We hypothesized that the excellent ROS scavenging capacity of H‐Cu_2_O preserves the Trx‐1 in its reduced state, thereby enforcing TXNIP sequestration and preventing NLRP3 activation.

qPCR analysis confirmed a redox‐induced molecular change. DSS stimulation increased *Nrf2* and *TXNIP* mRNA levels while reducing *Trx‐1* expression, indicating a prolonged oxidative stress state (Figure 5a–c). H‐Cu_2_O treatment normalized *Nrf2* and *Trx‐1* mRNA levels and significantly reduced *TXNIP* transcription and protein levels (Figure 5c,d).

**FIGURE 5:**
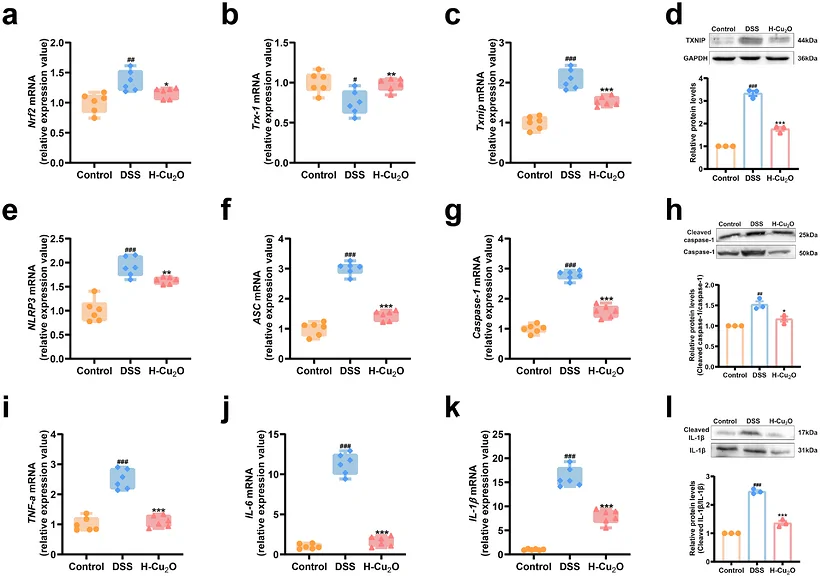
H‐Cu_2_O nanozymes suppress colonic inflammation by modulating the ROS‐TXNIP‐NLRP3 signaling axis. (a–c) Relative colonic mRNA expression levels of *Nrf2*, *Trx‐1*, and *TXNIP*. (d) Representative Western blot bands and corresponding densitometric quantification of the TXNIP protein. (e–g) Relative colonic mRNA expression levels of inflammasome components (*NLRP3*, *ASC*, and *Caspase‐1*). (h) Representative Western blot bands and densitometric quantification of cleaved Caspase‐1. (i–k) Relative colonic mRNA expression levels of downstream pro‐inflammatory cytokines (*TNF‐α*, *IL‐6*, and *IL‐1β*). (l) Representative Western blot bands and densitometric quantification of cleaved IL‐1β. Data are presented as mean ± SD. ^#^ *p* < 0.05, ^##^ *p* < 0.01, ^###^ *p* < 0.001 (*vs*. Control); ^*^ *p* < 0.05, ^**^ *p* < 0.01, ^***^ *p* < 0.001 (vs. DSS). Uncropped Western blot images are provided in Figures S33–S38.

Downstream of this regulatory node, DSS challenge triggered a marked transcriptional upregulation of the inflammasome components (*NLRP3*, *ASC*, and *Caspase‐1*), which was strongly suppressed by H‐Cu_2_O (Figure 5e–g). Western blot analysis showed H‐Cu_2_O effectively inhibited DSS‐induced cleaved caspase‐1 (Figure 5h), blocking inflammasome assembly. Consequently, H‐Cu_2_O normalized DSS‐elevated pro‐inflammatory cytokine mRNA such as *TNF‐α*, *IL‐6*, and *IL‐1β* (Figure 5i–k), and significantly reduced cleaved IL‐1β protein levels (Figure 5l). In addition, to causally test NLRP3 dependence in vitro, the selective NLRP3 inhibitor MCC950 pre‐treatment markedly reduced *NLRP3* mRNA expression. Critically, co‐treatment with H‐Cu_2_O and MCC950 produced no statistically significant additional reduction in *IL‐1β* mRNA compared with MCC950 alone (Figures S16 and S17). These results demonstrate that the anti‐inflammatory effect of H‐Cu_2_O on IL‐1β is dependent on a functional NLRP3 inflammasome.

These findings indicate that H‐Cu_2_O reduces mucosal ROS, maintaining the reduced Trx‐1 and inhibiting TXNIP. This redox adjustment prevents TXNIP from activating NLRP3, stops caspase‐1 cleavage, and blocks IL‐1β maturation, breaking the inflammatory cycle. Although genetic knockout studies are needed to confirm causality, the data are consistent with disruption of the ROS‐TXNIP‐NLRP3 axis as the primary anti‐inflammatory mechanism of H‐Cu_2_O.

### 2.6 H‐Cu_2_O Restores Gut Microbiota Homeostasis

Having established the anti‐inflammatory mechanism of H‐Cu_2_O, we next examined its effects on gut microbial homeostasis. Fecal microbial communities from the Control, DSS, and H‐Cu_2_O‐treated groups were analyzed by 16S rRNA gene sequencing. Microbial community alterations were evaluated using operational taxonomic unit overlap, principal coordinate analysis, taxonomic composition, and genus‐level differential abundance analysis [61, 62]. Although the core microbiome was largely retained, DSS exposure and H‐Cu_2_O treatment markedly altered the overall microbial community structure (Figure 6a). PCoA demonstrated that DSS‐treated communities diverged markedly from controls, whereas H‐Cu_2_O‐treated communities re‐aligned closely with control baselines, indicating ecological restoration (Figure 6b). Taxonomic evaluations at various levels confirmed community restructuring, with Lactobacillus showing significant changes at the genus level (Figure 6c–e). Its abundance decreased after DSS‐induced stress but increased with H‐Cu_2_O treatment, driving taxonomic differentiation (Figure 6f–h). In addition, PICRUSt2 functional prediction further confirmed that DSS‐associated depletion of metabolic pathways was significantly reversed by H‐Cu_2_O treatment (Figure S18). The intestinal ROS burden in colitis selectively pressures oxygen‐sensitive commensals. By scavenging these ROS, H‐Cu_2_O alleviates this selective pressure and allows Lactobacillus to re‐establish.

**FIGURE 6:**
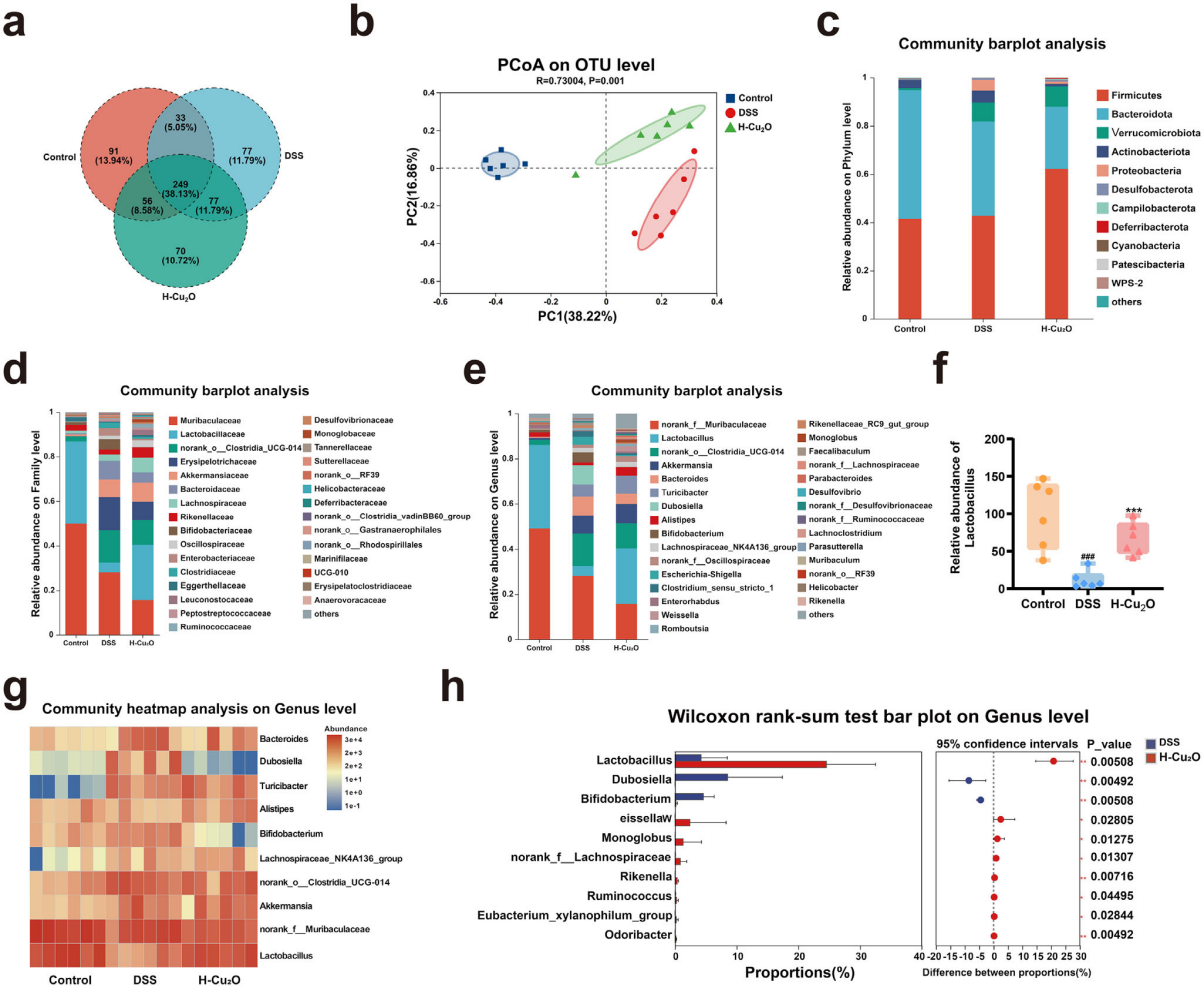
Gut microbiota remodeling following H‐Cu_2_O intervention. (a) Venn diagram illustrating operational taxonomic unit (OTU) overlaps among the Control, DSS, and H‐Cu_2_O groups. (b) Principal coordinate analysis (PCoA) at the OTU level demonstrating shifts in microbial community structure. (c–e) Stacked bar plots detailing the relative taxonomic abundances at the phylum (c), family (d), and genus (e) levels. (f) Quantitative analysis of the relative abundance of *Lactobacillus* across the experimental groups. (g) Differential abundance heatmap depicting the distribution patterns of dominant genera. (h) Differential genera analysis at the genus level (Wilcoxon rank‐sum test).

Given that this robust *Lactobacillus* enrichment serves as a quantifiable microbiological index of mucosal redox recovery, we selected *Lactiplantibacillus plantarum* (*L. plantarum*) HMPM2111 as a model probiotic to formulate a combinatorial therapeutic method. We hypothesize a sequential combinatorial mechanism: H‐Cu_2_O first neutralizes the hostile oxidative and inflammatory microenvironment, paving an ecological niche conducive to lactic acid bacteria colonization; subsequently, the exogenously supplemented *L. plantarum* fortifies microbial community stability and epithelial barrier function, yielding combinatorial therapeutic outcomes that surpass the efficacy of H‐Cu_2_O monotherapy [63, 64].

### 2.7 H‐Cu_2_O Alleviates Oxidative Pressure to Rescue *L. plantarum* Proliferation

We selected *L. plantarum* HMPM2111 as a model probiotic. The effects of H‐Cu_2_O on probiotic growth were evaluated by monitoring the growth kinetics of *L. plantarum* and *A. muciniphila* under normal and H_2_O_2_‐induced oxidative conditions. Sequential co‐administration of free *L. plantarum* and H‐Cu_2_O was further compared with the electrostatically assembled LP@H‐Cu_2_O hybrid using ex vivo fecal microbiota models, bacterial qPCR, and a Transwell‐ICP diffusion assay. As a representative of the *Lactobacillus* genus, it is consistently depleted during DSS‐induced dysbiosis, underscoring its ecological relevance [65, 66]. We sought to determine whether the potent ROS scavenging activity of H‐Cu_2_O could directly neutralize the oxidative pressure, thereby rescuing *L. plantarum* colonization in the inflamed gut.

Growth kinetic assays first confirmed the biocompatibility of the nanozymes. Exposure to therapeutic concentrations (25 and 50 µg mL^−1^) exerted no inhibitory effects on the proliferation of *Akkermansia muciniphila* (*A. muciniphila*) or *L. plantarum* under standard conditions (Figure 7a,b). However, under simulated oxidative stress (2 mm H_2_O_2_), *L. plantarum* proliferation was severely inhibited. The co‐administration of H‐Cu_2_O substantially reversed this suppression, rescuing bacterial growth to near‐normal levels in a dose‐dependent manner (Figure 7c). This directly demonstrates that H‐Cu_2_O efficiently removes microenvironmental H_2_O_2_, thereby shielding the probiotic from oxidative inhibition and confirming its capacity to alleviate the selective pressure against *Lactobacillus*. To establish a precise in vivo baseline for evaluating the combination therapy, an additional 16S rRNA sequencing specifically quantified the depletion of *Lactobacillus* in DSS‐treated murine fecal samples, confirming its significant decline at the genus level (Figure 7d,e) and providing a quantifiable microbiological endpoint for subsequent interventions.

**FIGURE 7:**
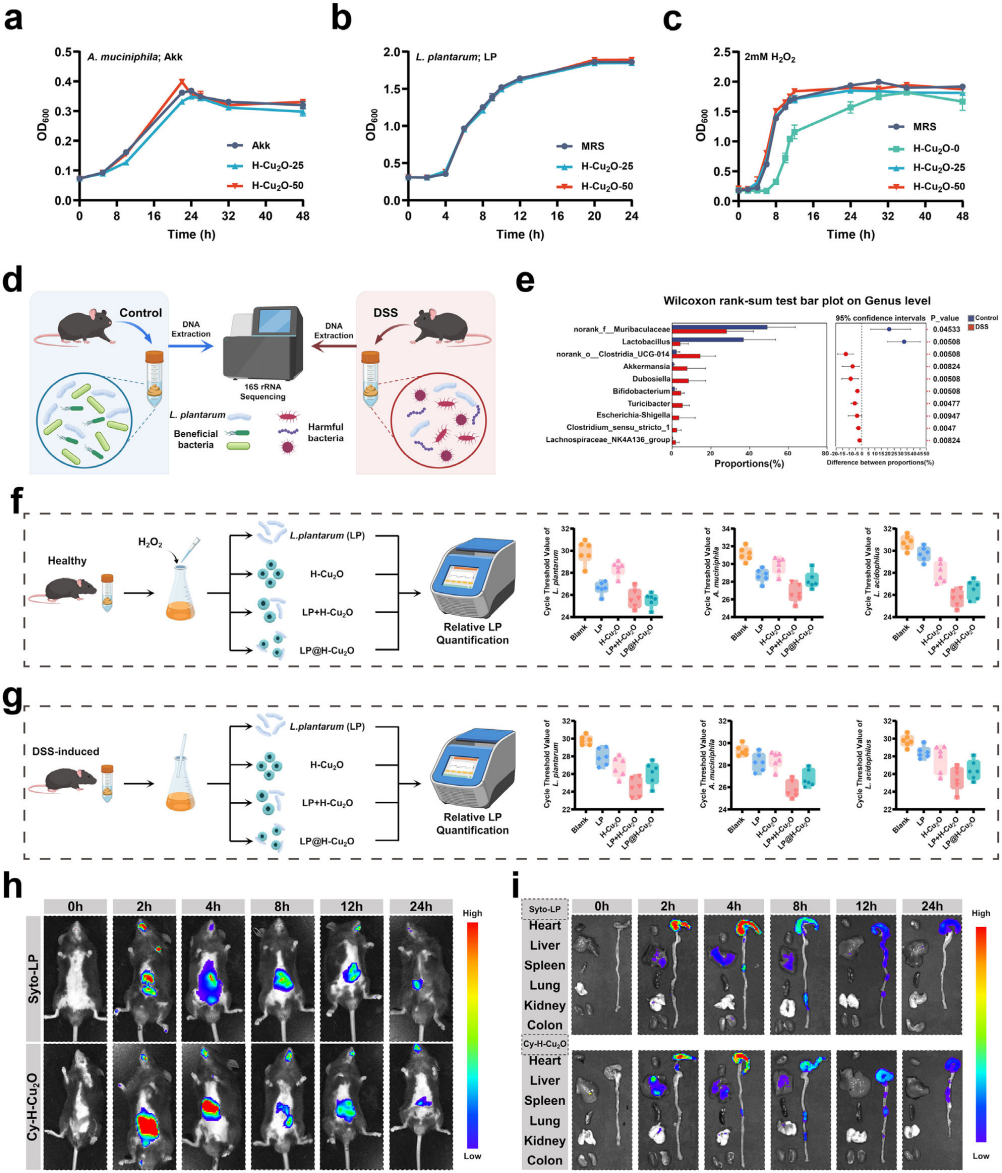
H‐Cu_2_O alleviates oxidative pressure to enhance *L. plantarum* proliferation and colonization. (a, b) Growth kinetics (OD_600 nm_) of *A. muciniphila* (a) and *L. plantarum* (b) cultured under standard conditions with H‐Cu_2_O (25 and 50 µg mL^−1^). (c) Growth kinetics demonstrating that H‐Cu_2_O mitigates the oxidative stress‐induced growth inhibition of *L. plantarum* under 2 mm H_2_O_2_ challenge (OD_600 nm_). (d) Schematic representation of the 16S rRNA sequencing workflow for murine fecal samples. (e) Genus‐level differential analysis revealing the significant depletion of *Lactobacillus* in the feces of DSS‐treated mice. (f, g) Schematic workflow and qPCR quantification. Healthy fecal microbiota + H_2_O_2_ challenge (f); DSS‐conditioned fecal microbiota (g); each with workflow schematic and qPCR of *L. plantarum*, *A. muciniphila*, and *L. acidophilus*. Fecal microbiomes from Control mice were challenged with H_2_O_2_, while those from DSS mice were used directly. Interventions included *L. plantarum*, H‐Cu_2_O, their sequential co‐administration (LP+H‐Cu_2_O), and the electrostatic hybrid (LP@H‐Cu_2_O). The box plots of cycle threshold (Ct) values (lower Ct indicates higher abundance) display the relative abundances. (h) In vivo fluorescence imaging illustrating the gastrointestinal transit and biodistribution over 24 h following oral co‐administration of Syto‐labeled *L. plantarum* and Cy‐labeled H‐Cu_2_O. (i) Ex vivo fluorescence imaging of major organs and the colon at specified time points, confirming intestinal retention and co‐localization. Data are presented as mean ± SD.

To optimize the delivery method, we compared a simple co‐administration (LP + H‐Cu_2_O) with an electrostatic adsorption hybrid (LP@H‐Cu_2_O; Figures S19 and S20). Growth kinetics monitoring confirmed that *L. plantarum* proliferation was not adversely affected under either delivery format (Figure S21), establishing comparable bacterial viability as a baseline for the subsequent comparison. Both were tested for protective efficacy against H_2_O_2_ in healthy and DSS‐induced fecal microbiota models (Figure 7f,g). qPCR analysis revealed that while both formats enhanced the survival of *L. plantarum* and key commensals (*A. muciniphila* and *Lactobacillus acidophilus*) relative to monotherapies, the co‐administration unexpectedly outperformed the electrostatically assembled LP@H‐Cu_2_O hybrid combination across multiple taxa.

To rigorously explain this functional difference, we performed a Transwell‐ICP diffusion experiment to directly quantify the transmembrane mobility of H‐Cu_2_O under each delivery format. NCM‐460 cells, pre‐challenged with DSS to simulate the inflamed colonic epithelium, were seeded in the lower compartment of a 1 µm pore‐size Transwell system, which permits free passage of H‐Cu_2_O nanoparticles while retaining bacteria. At both 25 and 50 µg mL^−1^, the LP + H‐Cu_2_O co‐administration delivered significantly greater amounts of H‐Cu_2_O to the lower‐compartment cells than the electrostatically assembled LP@H‐Cu_2_O complex, as quantified by intracellular copper content via ICP spectrometry (Figure S22). Consistently, qPCR analysis of the lower‐compartment NCM‐460 cells revealed that LP + H‐Cu_2_O treatment achieved significantly greater suppression of IL‐1β mRNA than LP@H‐Cu_2_O treatment (Figure S23), confirming that the enhanced nanozyme delivery translates into a measurable anti‐inflammatory advantage at the cellular level. This result directly demonstrates that electrostatic surface immobilization in LP@H‐Cu_2_O restricts nanozyme mobility, confining H‐Cu_2_O to the immediate microenvironment of the bacterial surface and limiting its access to distal epithelial and luminal targets. In contrast, freely dispersed H‐Cu_2_O in the sequential co‐administration diffuses throughout the intestinal milieu and reaches a broader range of cellular and microbial targets. The sequential co‐administration therefore preserves the functional independence of the bacterium while simultaneously allowing the nanozyme to distribute freely and scavenge ROS across the entire gut microenvironment, a spatial advantage that directly translates into the superior broad‐spectrum commensal protection observed across *L. plantarum*, *A. muciniphila*, and *L. acidophilus* in the fecal microbiota protection model (Figure 7g). Given this superior broad‐spectrum microbiota protective effect, formulation simplicity, and mechanistic clarity, the sequential co‐administration was selected for in vivo validation.

Finally, in vivo and ex vivo fluorescence imaging provided crucial spatiotemporal evidence for the synchronized gastrointestinal transit of the sequential co‐administration. Following oral gavage, Syto‐labeled *L. plantarum* and Cy‐labeled H‐Cu_2_O generated prominent, co‐localized gastrointestinal signals within 2–8 h, which remained detectable at 24 h (Figure 7h). To substantiate these observations quantitatively, semi‐quantitative ROI analysis was performed on all IVIS images using the Living Image software. Syto‐LP and Cy‐H‐Cu_2_O exhibited parallel signal kinetics in the gastrointestinal tract, both peaking at 4–8 h and persisting at 24 h, whereas free Syto and Cy dyes showed substantially lower and more rapidly clearing signals (Figure S24). Subsequent ex vivo imaging and quantification confirmed that fluorescence at 8–12 h was predominantly localized to the colon, with negligible signal in other major organs (Figure 7i and Figure S24e–h), demonstrating synchronized colonic co‐delivery of *L. plantarum* and H‐Cu_2_O. These data demonstrate that the sequential co‐administration achieves effective colonic co‐delivery within a shared therapeutic window, without requiring the electrostatic adsorption hybrid combination, combining favorable biocompatibility with translational practicality for combinatorial IBD nanotherapy.

This study adds to the debate in the probiotic‐nanozyme field on optimal binding modes for co‐delivery. Previous research often uses surface‐coating or encapsulation to attach nanozymes to bacteria for targeted mucosal delivery, concentrating activity but limiting spatial freedom. This work shows that for broad‐spectrum protection of the luminal microbiome, simple co‐administration, which preserves spatial freedom, is more effective than surface immobilization. Thus, surface‐conjugated systems are best for targeted mucosal delivery, while free co‐administration is better for wide gut coverage.

### 2.8 Combinatorial Therapeutic Efficacy of H‐Cu_2_O and *L. plantarum* Co‐Administration

To evaluate the combinatorial therapeutic efficacy, DSS‐treated mice were orally administered *L. plantarum*, H‐Cu_2_O, or their sequential combination (Figure 8a). Therapeutic efficacy was assessed by body weight, disease activity index, colon length, histopathology, inflammatory cytokine expression, and epithelial tight‐junction integrity. Systemic biosafety was evaluated by organ morphology, organ indices, histological examination, hemolysis assays, and tissue copper accumulation (Figure 8a). While monotherapies with either *L. plantarum* or H‐Cu_2_O partially ameliorated body weight loss, the disease activity index (DAI), and colon shortening, the sequential co‐administration consistently achieved superior rescue across all three macroscopic endpoints (Figure 8b,c and Figures S25 and S26). This confirms that the combinatorial strategy engages complementary therapeutic mechanisms. Histopathological analyses further suggested that the DSS‐induced mucosal disruption, crypt ablation, and massive inflammatory infiltration were most effectively reversed by the co‐administration, restoring the tissue architecture to a near‐healthy phenotype (Figure 8d). PAS staining similarly demonstrated that goblet cell depletion and mucus layer damage, which were only partially mitigated by monotherapies, were comprehensively restored by the sequential co‐administration (Figure 8e).

**FIGURE 8:**
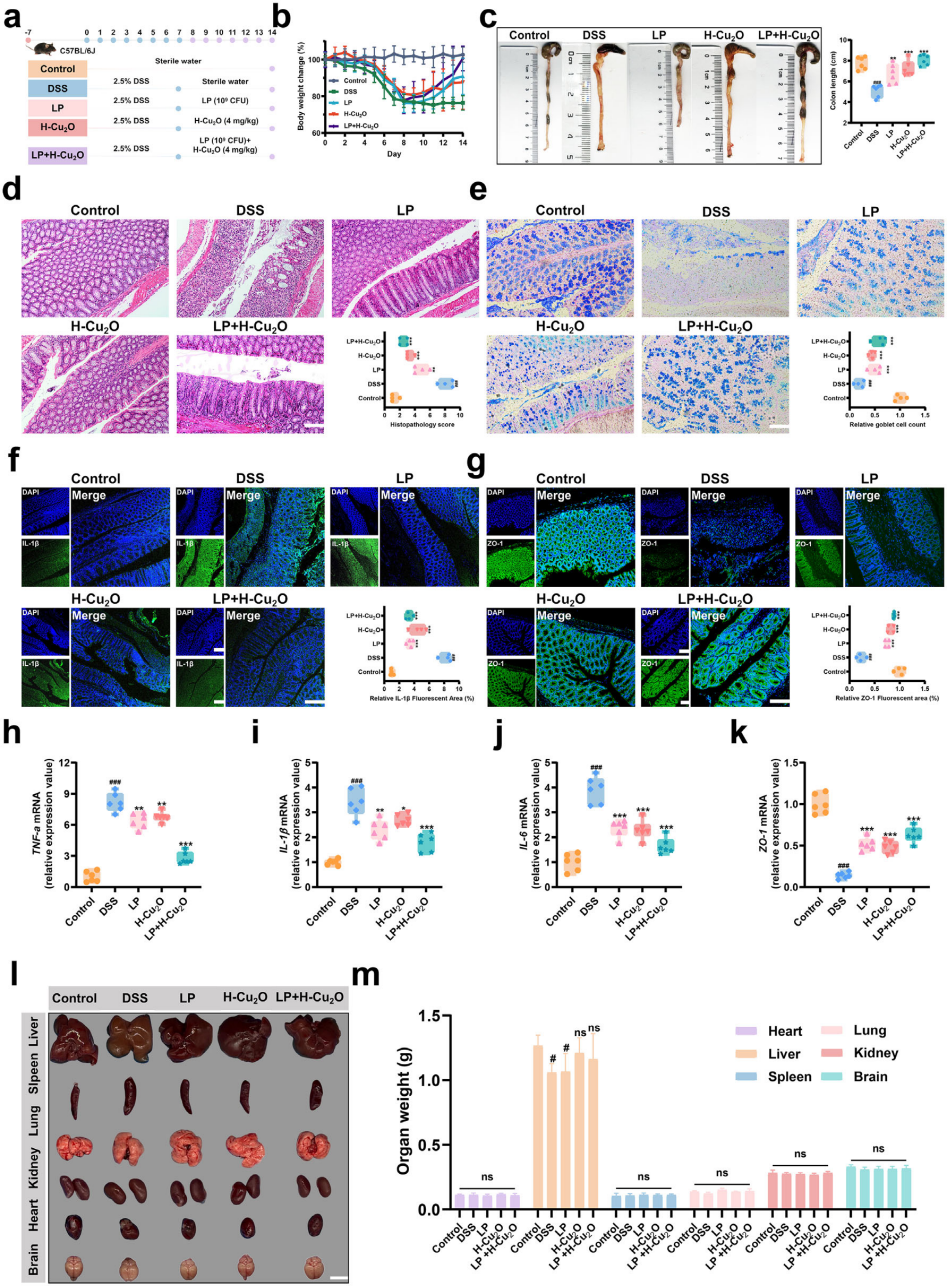
In vivo combinatorial therapeutic efficacy and biosafety of the H‐Cu_2_O + *L. plantarum* sequential co‐administration in DSS‐induced colitis. (a) Schematic representation of the combinatorial treatment protocol (DSS: 2.5%; *L. plantarum*: 10^9^ CFU/mouse; H‐Cu_2_O: 4 mg kg^−1^; Mixture: oral gavage of *L. plantarum* and H‐Cu_2_O sequential co‐administration). (b) Percentage change in body weight during the treatment period. (c) Representative macroscopic colon images and corresponding length quantification. (d) Representative H&E staining images and histopathological scoring (scale bar: 50 µm). (e) Representative PAS staining images and quantification of goblet cells (scale bar: 50 µm). (f) Immunofluorescence staining for IL‐1β protein and quantification of the IL‐1β‐positive area (scale bars: 50 µm). (g) Immunofluorescence staining for ZO‐1 protein and quantification of the ZO‐1‐positive area (scale bars: 50 µm). (h–k) Relative colonic mRNA expression levels of *TNF‐α*, *IL‐1β*, *IL‐6*, and *ZO‐1*. (l) Representative gross morphological images of major organs (scale bar: 10 mm). (m) Quantification of major organ weights. Data are presented as mean ± SD. ^#^ *p* < 0.05, ^##^ *p* < 0.01, ^###^ *p* < 0.001 (*vs*. Control); ^*^ *p* < 0.05, ^**^ *p* < 0.01, ^***^ *p* < 0.001 (vs. DSS).

At the molecular level, the combination therapy produced the greatest suppression of colonic IL‐1β protein (Figure 8f) and the most continuous ZO‐1 immunofluorescence at the epithelial boundary (Figure 8g). Quantitative analysis confirmed maximal reduction in IL‐1β‐positive area and optimal restoration of ZO‐1‐positive area. The mRNA levels further showed the combination therapy most comprehensively suppressed *TNF‐α*, *IL‐1β*, and *IL‐6* while most effectively restoring *ZO‐1*, *Occludin*, and *Claudin‐1* expression (Figure 8h–k and Figures S27 and S28).

Throughout the treatment duration, comprehensive safety assessments revealed no discernible abnormalities in gross organ morphology or organ weight distributions (Figure 8l,m). The copper content in the liver and kidney of H‐Cu_2_O‐treated mice was not significantly different from that of control mice, and the same was observed for the combination treatment group (Figures S30 and S31). Furthermore, histological evaluations of major organs, organ indices, and hemolysis assays (Figures S29 and S32) confirmed the excellent systemic biosafety and tolerability of the combination treatment at the effective therapeutic dosage. It should be noted that the present biosafety evaluation is limited to the 14‐day treatment window employed in this study, and long‐term safety under chronic dosing conditions remains to be formally established. Future work incorporating 16S rRNA profiling of the combination groups will be needed to directly quantify the microbiota level advantages of the LP + H‐Cu_2_O sequential co‐administration relative to individual treatments.

The combination therapy reflects the convergence of two mechanistically non‐overlapping pathways. H‐Cu_2_O provides an abiotic catalytic function, scavenging mucosal ROS to suppress the TXNIP/NLRP3 axis and generating a low oxidative stress microenvironment. Concurrently, *L. plantarum* activates biotic pathways that reinforce gut microbiota homeostasis and accelerate tissue repair. Together, these actions produce anti‐inflammatory and barrier‐repairing effects that exceed either monotherapy.

## 3 Conclusion

In summary, using composition‐matched solid Cu_2_O as a morphological control, we show that the transition to a hollow architecture markedly enhances broad‐spectrum ROS‐scavenging kinetics, as demonstrated by in vitro enzyme‐mimicking assays and BET characterization. This improvement stems from the exposure of interior active surfaces and the shortening of substrate diffusion paths. At the molecular level, H‑Cu_2_O‑mediated ROS clearance preserves the reduced Trx‑1/TXNIP inhibitory complex, thereby blocking TXNIP‑driven NLRP3 inflammasome assembly and subsequent caspase‑1‑dependent IL‑1β maturation. The abiotic redox regulation from H‑Cu_2_O alleviates the selective pressure on oxygen‐sensitive gut commensals, thereby guiding the dysbiotic gut microbiota toward a homeostatic state marked by an enrichment of Lactobacillus species. Notably, the simple sequential co‑administration of *L. plantarum* and H‑Cu_2_O outperforms the electrostatically assembled LP@H‐Cu_2_O hybrid combination. The functional independence and free nanozyme diffusivity within the gut microenvironment are more effective than electrostatic surface immobilization for achieving nanozyme probiotic therapeutic synergy. Future studies should validate the causal TXNIP dependence using genetic knockout models and assess long‐term gastrointestinal biodistribution.

## 4 Experimental Section

### 4.1 Materials

*Lactiplantibacillus plantarum* (*L. plantarum*) HMPM2111 was provided by the Anhui Provincial Key Laboratory of Human Microecology and Precision Medicine (Anhui University) and is deposited at the Guangdong Microbial Culture Collection Centre (GDMCC No. 64520). Human normal colonic epithelial cells (NCM‐460; INCELL Corporation; product code NCM460D; RRID: CVCL_0460) and murine macrophages (RAW264.7; Cell Bank of the Chinese Academy of Sciences; original ATCC source TIB‐71; RRID: CVCL_0493) were utilized for in vitro experiments.

7‐week‐old specific‐pathogen‐free (SPF) male C57BL/6J mice (body weight 20 ± 2 g) were purchased from Hefei Qingyuan Biotechnology Co., Ltd. (Hefei, China). Animals were maintained under standard SPF conditions with a 12 h light/dark cycle and ad libitum access to standard chow and sterilized water. All animal procedures were approved by the Institutional Animal Care and Use Committee of Anhui University (No. IACUC(AHU)‐2025‐029) and conducted in full compliance with the ARRIVE 2.0 guidelines for reporting animal experiments.

Growth media components (yeast extract, peptone, beef extract, and glucose) were purchased from Sangon Biotech (Shanghai, China). Staining and analytical reagents included DAPI, anti‐fade mounting medium, and a GSH/GSSG detection kit (Beyotime Biotechnology, Shanghai, China); DPPH, ABTS, 3,3′,5,5′‐tetramethylbenzidine (TMB), and H_2_O_2_ (Shanghai Yuanye Bio‐Technology, Shanghai, China); ALT and AST activity kits (Nanjing Jiancheng Bioengineering Institute, Nanjing, China); dextran sulfate sodium (DSS; MW 36 000–50 000; Beijing Cybrant Technology, Beijing, China); and enzymatic activity/content kits for MDA, MPO, SOD, and CAT (Nanjing Jiancheng Bioengineering Institute, Nanjing, China). An Alcian Blue‐PAS (AB‐PAS) staining kit was obtained from Shanghai Jizhi Biological Technology (Shanghai, China).

Molecular and protein analysis reagents included Hieff qPCR SYBR Green Master Mix and Hifair II First‐Strand cDNA Synthesis SuperMix (YEASEN Biotechnology, Shanghai, China), alongside routine reagents for Western blotting and biomolecular extraction (BCA protein assay kit, ECL detection reagent, TRIzol RNA extraction reagent, RIPA lysis buffer, PMSF, PVDF membranes, pre‐stained protein markers, and rapid electrotransfer buffer). Primary antibodies recognizing the following targets were employed: Occludin, Claudin‐1, TXNIP, ASC (Abcam, Shanghai, China); Caspase‐1/cleaved Caspase‐1, IL‐1β/cleaved IL‐1β (Cell Signaling Technology, Shanghai, China); and ZO‐1, GAPDH (loading control) (Proteintech, Wuhan, China). All chemical reagents (including CuCl_2_·2H_2_O, FeSO_4_, Ti(SO_4_)_2_, HCl, NaOH, NaCl, and KCl) were of analytical grade. All solutions were prepared using ultrapure deionized water.

### 4.2 Synthesis of Cu_2_O Nanozymes

#### 4.2.1 Synthesis of Hollow Cuprous Oxide Nanozymes (H‐Cu_2_O)

Poly(vinylpyrrolidone) (PVP; K30, MW ≈ 40 000; 0.4 g) was dissolved in anhydrous ethanol (30 mL) and stirred for 20 min. Subsequently, CuCl_2_·2H_2_O (0.17 g) was added and stirred for 30 min. At room temperature, hydrazine hydrate (N_2_H_4_·H_2_O, 80%; 1 mL) was added dropwise as the reducing agent under rapid stirring (600 rpm) for a further 60 min. The resulting suspension was transferred to an autoclave (50 mL) and treated at 100°C for 120 min. After cooling naturally to room temperature, the orange‐brown precipitate was collected by centrifugation (8000 rpm, 5 min), washed three times with deionized water and twice with ethanol, and then vacuum‐dried at 60°C for 4 h.

#### 4.2.2 Synthesis of Solid Cuprous Oxide Nanozymes (S‐Cu_2_O)

The synthetic route for S‐Cu_2_O was essentially the similar as that for H‐Cu_2_O, with two key adjustments made to kinetically suppress the formation of internal cavities: (i) the amount of PVP was increased to 0.5 g for enhancing steric stabilization and promoting the formation of dense particles; (ii) during the dropwise addition of hydrazine hydrate at room temperature, slow stirring (300 rpm) was applied, and stirring was continued for 60 min after the addition was complete. The mixture was then transferred to an autoclave and treated at 100°C for 180 min. All other parameters, including reagent concentrations, solvent volume, amount of reducing agent, and washing and drying steps, were kept identical to those used for H‐Cu_2_O.

### 4.3 Materials Characterization

Powder X‐ray diffraction (XRD) patterns were collected using Cu Kα radiation (λ = 0.15406 nm) on a SmartLab 9 kW diffractometer (Rigaku, Japan) over a 2θ range of 20–80° at a scan rate of 5° min^−1^. Transmission electron microscopy (TEM) images were acquired on a JEM‐2100 instrument (JEOL, Japan) operating at 200 kV. Fourier‐transform infrared (FTIR) spectra were recorded on a Nicolet iS50 spectrometer (Thermo Fisher Scientific, USA) in ATR mode over 4000–500 cm^−1^ with a resolution of 4 cm^−1^ (32 co‐added scans). X‐ray photoelectron spectroscopy (XPS) analyses were performed on an ESCALAB 250Xi spectrometer (Thermo Fisher Scientific, USA) equipped with a monochromatic Al Kα X‐ray source (hν = 1486.6 eV). Survey spectra were acquired at a pass energy of 100 eV; high‐resolution Cu 2p and O 1s spectra were collected at 20 eV. Binding energies were calibrated referencing the adventitious C 1s peak at 284.8 eV. Cu LMM Auger spectra were acquired under identical conditions for the unambiguous assignment of the Cu oxidation states. Peak deconvolution was performed using CasaXPS (v2.3.24) incorporating a Shirley background subtraction.

### 4.4 Bacterial Growth Experiments and Preparation of the Electrostatic Adsorption Hybrid (LP@H‐Cu_2_O)

#### 4.4.1 Growth Kinetics of *L. plantarum* and *A. muciniphila*

Activated *L. plantarum* HMPM2111 was inoculated (1% v/v) into fresh MRS broth containing H‐Cu_2_O (0, 25, or 50 µg mL^−1^). Cultures were incubated at 37°C (200 rpm), and the optical density (OD_600 nm_) was monitored over 24 h. Viability was further verified via spot‐plating serial dilutions onto MRS agar plates. For *A. muciniphila*, culturing was performed in Brain Heart Infusion (BHI) broth supplemented with 0.1% (w/v) mucin under strict anaerobic conditions (N_2/_CO_2_ = 80:20). H‐Cu_2_O (0, 25, or 50 µg mL^−1^) was added to pre‐reduced media, and the OD_600 nm_ was tracked over 48 h.

#### 4.4.2 Bacterial Growth Under Oxidative Challenge

To demonstrate H‐Cu_2_O‐mediated oxidative protection, *L. plantarum* was inoculated into MRS broth under four conditions: (i) Control (no additives); (ii) H_2_O_2_ challenge (2 mM); (iii) H_2_O_2_ + H‐Cu_2_O (25 µg mL^−1^); and (iv) H_2_O_2_ + H‐Cu_2_O (50 µg mL^−1^). OD_600 nm_ values were recorded over predetermined intervals. All conditions were evaluated in biological triplicate.

#### 4.4.3 Preparation of the Electrostatic Adsorption Hybrid (LP@H‐Cu_2_O)

*L. plantarum* was cultured to an OD_600 nm_ of 1.6, and the suspension was adjusted to 5 × 10^9^ CFU mL^−1^. An H‐Cu_2_O stock suspension (10 mg mL^−1^) was dispersed via pulsed probe sonication. The bacterial suspension was washed twice with sterile PBS and resuspended in sterile deionized water. The H‐Cu_2_O stock was added to achieve a final nanozyme concentration of 25 µg mL^−1^ in a 5 mL reaction volume. The mixture was incubated at 4°C on a 360° rotator for 3 h to facilitate electrostatic adsorption. The resulting LP@H‐Cu_2_O complexes were collected via centrifugation (5000 rpm, 8 min), resuspended in sterile water, and utilized within 24 h.

### 4.5 In Vivo Murine Colitis Model and Combinatorial Intervention

#### 4.5.1 Preparation of the *L. plantarum* Inoculum

*L. plantarum* HMPM2111 was revived from −80°C glycerol stocks in fresh, sterile MRS broth and cultured overnight at 37°C with shaking. The strain was purified via repeated streaking and single‐colony expansion on MRS agar plates to ensure strain homogeneity. For oral gavage, bacterial suspensions were concentrated and adjusted to the required dosage based on colony‐forming unit (CFU) enumeration.

#### 4.5.2 DSS‐Induced Colitis and Combinatorial Therapy

Following a 1‐week acclimatization period, C57BL/6J mice were randomly divided into five groups (*n* = 6/group): Control, DSS, *L. plantarum* (LP), H‐Cu_2_O, and *L. plantarum* + H‐Cu_2_O (LP+H‐Cu_2_O). The Control group received ad libitum sterile water. Acute colitis was induced in the remaining groups via the administration of DSS (2.5% w/v) in the drinking water for 7 consecutive days, followed by a 7‐day recovery phase receiving sterile water. During this recovery phase, mice received daily therapeutic interventions via oral gavage: the *L. plantarum* group received 1 × 10^9^ CFU/mouse; the H‐Cu_2_O group received 4 mg kg^−1^ nanozyme; and the combinatorial group received *L. plantarum* (1 × 10^9^ CFU) and H‐Cu_2_O (4 mg kg^−1^) administered sequentially with a 30‐min interval. At the experimental endpoint, mice were fasted for 5–6 h, anesthetized, and euthanized. Serum, colon, liver, and fecal samples were systematically harvested, weighed, and either fixed in tissue fixative or snap‐frozen at −80°C.

### 4.6 Statistical Analysis

Data are presented as mean ± standard deviation (SD). Statistical analyses were performed using GraphPad Prism (version 8.0). Two‐group comparisons were conducted using Student's *t*‐test, and multiple‐group comparisons were analyzed by one‐way ANOVA with appropriate multiple‐comparison correction. Genus‐level differential abundance was analyzed by the Wilcoxon rank‐sum test. A *p*‐value < 0.05 was considered statistically significant. Significance was denoted as ^#^ *p* < 0.05, ^##^ *p* < 0.01, ^###^ *p* < 0.001 vs. the control group, ^*^ *p* < 0.05, ^**^ *p* < 0.01, ^***^ *p* < 0.001 versus the model group, with exact comparisons defined in figure legends.

## Author Contributions

K.S., S.G., and Y.W. conceived and designed this study. J.C., G.W., P.L., Y.W., and S.L. performed the experiments. J.C., G.W., and P.L. conducted the data analysis and prepared figures and tables. G.W., Y.W., and S.L. wrote the manuscript. All authors reviewed and approved the manuscript.

## Conflicts of Interest

The authors declare no conflicts of interest.

## Supporting information




**Supporting File**: advs76598‐sup‐0001‐SuppMat.docx.

## Data Availability

The data that support the findings of this study are available from the corresponding author upon reasonable request.
