# Supplementary material for: Hollow Cu2O Nanozymes Enhance Probiotic Therapy for Colitis via Redox Homeostasis and TXNIP/NLRP3 Inflammasome Inhibition
Source: Adv Sci (Weinh). 2026 Jul 14:e76598. Online ahead of print. doi: 10.1002/advs.76598 (PMC13367104; doi:10.1002/advs.76598)
Supplement: Supplementary file 1 — Supporting File: advs76598‐sup‐0001‐SuppMat.docx [file ADVS-9999-e76598-s001.docx]

Supporting Information

**Hollow Cu_2_O Nanozymes Enhance Probiotic Therapy for Colitis via Redox Homeostasis and TXNIP/NLRP3 Inflammasome Inhibition**

Guangzhao Wang^1,2^, Jialing Cao^1,2^, Pengfei Li^1,2^, Yujie Wang^1,2^, Shiqi Lu^1,2^, Kangliang Sheng^1,2*^, Shan Gao^3*^, Yongzhong Wang^1,2*^

^1^School of Life Sciences and Medical Engineering, Anhui University, Hefei 230601, Anhui, China.

^2^Key Laboratory of Human Microenvironment and Precision Medicine of Anhui Higher Education Institutes, Anhui University, Hefei 230601, Anhui, China.

^3^School of Chemistry and Chemical Engineering, Anhui University, Hefei 230601, Anhui, China.

* Corresponding author:

Kangliang Sheng, E-mail: [kangliang@ahu.edu.cn](mailto:kangliang@ahu.edu.cn)

Shan Gao, E-mail: shangao@ahu.edu.cn

Yongzhong Wang, E-mail: yzwang@ahu.edu.cn

**Supplementary Experimental Section**

**ROS Scavenging and Enzyme-Mimicking Assays**

**CAT-like activity and H_2_O_2_ scavenging:** Catalase (CAT)-mimicking activity and residual H_2_O_2_ concentrations were quantified using an ammonium molybdate colorimetric assay kit (Nanjing Jiancheng). Briefly, nanozymes (25-250 µg mL^-1^) were co-incubated with H_2_O_2_ (20 mM, pH 7.4) at 37 °C for 2 h under gentle agitation. After the addition of stop solution and chromogenic reagent, absorbance was measured at 405 nm. Scavenging efficiency (%) = [1 − (A_sample_ − A_blank_) / (A_control_ − A_blank_)] × 100%.

**O_2_•^-^ scavenging:** Superoxide anions (O_2_•^-^) were generated in situ using a xanthine/xanthine oxidase system. Nanozymes (25-300 µg mL^-1^) were introduced into a reaction mixture containing xanthine (0.1 mM) and xanthine oxidase (0.1 U mL^-1^) in sodium carbonate buffer (50 mM, pH 10.2), followed by incubation at 37 °C for 40 min. Ascorbic acid (1 mM) served as the positive control. After addition of the Griess chromogenic reagent, absorbance was measured at 550 nm. Scavenging efficiency (%) = [1 − (A_sample_ / A_control_)] × 100%.

**OH• scavenging:** Hydroxyl radicals (OH•) were generated by the Fe^2+^/H_2_O_2_ Fenton reaction and quantified using a TMB colorimetric assay. In sodium acetate buffer (0.1 M, pH 5.5), nanozymes (25-250 µg mL^-1^) were incubated with FeSO_4_ (0.2 mM) and H_2_O_2_ (0.2 mM) at 37 °C for 15 min. TMB (0.2 mM) was then added, and absorbance at 510 nm was recorded immediately.

**DPPH• and ABTS•+ scavenging:** DPPH• scavenging was evaluated by mixing nanozymes (25-250 µg mL^-1^) with a DPPH• ethanol solution (0.04 mg mL⁻¹) at a 1:1 (v/v) ratio, followed by incubation in the dark at room temperature for 30 min before absorbance was measured at 517 nm. For ABTS•+ scavenging, an ABTS•+ working solution, pre-oxidized by K_2_S_2_O_8_ and diluted to an absorbance of 0.750 ± 0.025 at 734 nm, was co-incubated with nanozymes for 10 min, and absorbance was recorded at 734 nm. Scavenging efficiencies were calculated relative to the corresponding reagent-only controls.

**Dissolved O_2_ generation kinetics:** To monitor CAT-like activity in real time, H-Cu_2_O or S-Cu_2_O (100-250 µg mL^-1^) was added to an H_2_O_2_ solution (20 mM, pH 7.4) in a sealed chamber. Dissolved oxygen (DO) concentration was continuously recorded using a DO electrode (PreSens, Germany) at 37 °C for 10 min. Initial O_2_ evolution rates were extrapolated from the linear region of the DO-time curves (0-2 min).

**Catalase-like kinetic analysis:** The catalase-like kinetics of Cu_2_O nanozymes were evaluated using H_2_O_2_ as the substrate. Cu_2_O nanozymes were added to the reaction system at a final concentration of 100 µg mL^-1^ and incubated with H_2_O_2_ at concentrations of 0, 0.02, 0.04, 0.06, 0.08, 0.10, and 0.12 mol L^-1^. The initial reaction velocity was calculated from the linear range of absorbance change. Michaelis-Menten fitting was performed to determine the apparent kinetic parameters, including the maximum reaction velocity (Vmax) and Michaelis constant (Km), according to v = Vmax[S]/(Km + [S]). Lineweaver-Burk double-reciprocal plots were used as a complementary validation of kinetic fitting. The 0 mol L^-1^ H_2_O_2_ group was excluded from double-reciprocal analysis.

**SOD-like activity assay:** The SOD-like activity of Cu_2_O nanozymes was determined using a commercial superoxide dismutase assay kit (Nanjing Jiancheng Bioengineering Institute, China) according to the manufacturer’s protocol. Briefly, Cu_2_O nanozymes were prepared at concentrations of 5, 10, 25, 50, 100, 200, and 400 µg mL^-1^, followed by sequential reagent addition, incubation, and colorimetric detection. Absorbance was recorded, and the corresponding SOD-like activity was calculated according to the kit instructions. The concentration-dependent activity curve was generated by plotting nanozyme concentration against enzymatic activity.

**Environmental tolerance:** To assess catalytic robustness, the H_2_O_2_ scavenging efficiency of nanozymes (250 µg mL^-1^) was profiled across different temperatures (20-100 °C) and pH values (pH 4-12). Results were normalized to standard physiological conditions (25 °C or pH 7.4) and expressed as relative activity (%).

***In Vitro* Cell Culture and Biological Assays**

**Cell culture:** Human normal colonic epithelial cells (NCM-460; INCELL Corporation; product code NCM460D; RRID:CVCL_0460) were cultured in high-glucose Dulbecco’s Modified Eagle Medium (DMEM) supplemented with 10% fetal bovine serum (FBS) and 1% penicillin-streptomycin. Murine macrophages (RAW264.7; Cell Bank of the Chinese Academy of Sciences; original ATCC source TIB-71; RRID:CVCL_0493) were maintained in complete RAW264.7-specific medium. All cell lines were incubated at 37 °C in a humidified atmosphere containing 5% CO_2_.

**Cytotoxicity assay (CCK-8):** Cells were seeded in 96-well plates (8000 cells/well) and incubated for 24 h. H-Cu_2_O nanozymes were then added and co-incubated for 12 or 24 h. CCK-8 working solution was added and incubated for an additional 3 h. Absorbance was measured at 450 nm using a microplate reader. Cell viability was expressed as a percentage relative to the untreated control.

**H_2_O_2_-Induced oxidative stress model:** NCM-460 cells were seeded in 96-well plates and cultured for 12 h. Oxidative stress was induced by adding predetermined concentrations of H_2_O_2_, followed by concurrent treatment with or without H-Cu_2_O (0, 25, or 50 µg mL^-1^; n = 3 wells/condition). After incubation, the medium was replaced with fresh complete medium to eliminate potential optical interference before the CCK-8 assay was performed as described above.

**Intracellular ROS imaging:** NCM-460 cells were seeded in 6-well plates. After adhesion, cells were stimulated with 400 µM H_2_O_2_ for 12 h. The medium was then replaced with fresh medium containing H-Cu_2_O (25 or 50 µg mL^-1^), and cells were incubated for another 12 h. After treatment, cells were incubated with the DCFH-DA fluorescent probe diluted in serum-free medium at 37 °C for 20 min. After washing three times with PBS to remove excess probe, intracellular ROS levels were visualized using a fluorescence microscope. Mean fluorescence intensity was quantified using ImageJ software.

**Anti-Inflammatory interventions:** For the RAW264.7 macrophage model, cells seeded in 6-well plates were challenged with lipopolysaccharide (LPS; 0.5 µg mL^-1^) for 12 h. After LPS stimulation, the medium was removed, and cells were incubated with fresh medium containing H-Cu_2_O (25 or 50 µg mL^-1^) for another 12 h. Supernatants were collected for mouse IL-18, IL-1β, and TNF-α ELISA, and cell pellets were processed for RNA extraction and qRT-PCR detection of the corresponding cytokines.

**Cellular uptake assay:** Rhodamine B-labeled hollow cuprous oxide (H-Cu_2_O) nanozymes were synthesized using an EDC/NHS-assisted labeling strategy. Briefly, 170 mg EDC, 100 mg NHS, and 100 mg H-Cu_2_O nanozymes were sequentially dispersed in 10 mL double-distilled water by ultrasonication, followed by stirring at room temperature for 1 h. Subsequently, 50 μmol NH₂-Rhodamine B was added, and the reaction was allowed to proceed for 48 h under light-protected conditions. The resulting mixture was transferred to a dialysis bag (MWCO 1000 kDa) and dialyzed against double-distilled water in the dark for 12 h, with the external dialysis solution refreshed every 2 h to remove unconjugated dye. After dialysis, the nanozyme suspension was further washed and centrifuged to eliminate residual free dye. The obtained RhB-labeled H-Cu_2_O nanozymes were stored in the dark until use.

For uptake assays, NCM460 cells were seeded in confocal dishes or glass-bottom dishes and allowed to adhere. Two separate experiments were performed. For the time-dependent study, cells were incubated with RhB-labeled H-Cu_2_O nanozymes at a fixed concentration of 50 µg mL^-1^ for 0, 2, 6, 12, and 24 h at 37 °C. For the concentration-dependent study, cells were treated with nanozymes at final concentrations of 0, 10, 25, and 50 µg mL^-1^ for 12 h at 37 °C. After incubation, cells were gently washed three times with PBS to remove non-internalized or loosely attached nanomaterials. Cell membranes were stained with DiO (green), and nuclei were counterstained with DAPI. Fluorescence images were captured using a confocal laser scanning microscope to assess time- and concentration-dependent cellular uptake of H-Cu_2_O nanozymes.

**NLRP3 pathway validation:** After RAW264.7 cell culture, cells were divided into five groups: Control, DSS model, MCC950, hollow cuprous oxide (H-Cu_2_O), and combined treatment . Cells in the Control group were maintained under normal culture conditions. In the DSS model group, cells were exposed to 5% DSS (w/v) for 12 h to induce an inflammatory injury model. In the MCC950 group, cells were pretreated with 5 μM MCC950 for 1 h, followed by co-incubation with 5% DSS (w/v) for 12 h. In the H-Cu_2_O group, cells were pretreated with 50 µg mL^-1^ H-Cu₂O for 1 h and then co-incubated with 5% DSS (w/v) for 12 h. In the combined treatment group, cells were simultaneously pretreated with 5 μM MCC950 and 50 µg mL^-1^ H-Cu_2_O for 1 h, followed by exposure to 5% DSS (w/v) for 12 h. MCC950 was dissolved in DMSO to prepare a stock solution and diluted with complete culture medium to a final concentration of 5 μM immediately before use. The final DMSO concentration was kept below 0.1%, and an equal volume of DMSO was added to the Control group as the vehicle control. H-Cu_2_O was diluted to the indicated working concentration with complete culture medium. After treatment, culture supernatants and cell lysates were collected for subsequent analysis of inflammatory mediators and related protein expression.

**Diffusion efficiency of LP + H-Cu_2_O and LP@H-Cu_2_O:** A Transwell assay was performed to evaluate the effect of different binding modes between H-Cu_2_O nanozymes and the probiotic LP on transmembrane diffusion. NCM460 cells were seeded in the lower compartment of a Transwell system (pore size: 1 μm) and allowed to adhere. After stabilization, an *in vitro* inflammatory model was established by treating cells with 5% DSS (w/v) for 12 h. Two intervention forms were then added to the upper compartment: a simple mixture of H-Cu_2_O nanozymes and LP (LP + H-Cu_2_O) and an electrostatically assembled complex (LP@H-Cu_2_O). For each form, H-Cu_2_O was applied at final concentration gradients of 25 and 50 µg mL^-1^. After an additional 12 h incubation, the upper insert was removed, and the cells in the lower compartment were gently washed with PBS, collected, and centrifuged. The cell pellet was resuspended in 500 μL PBS, subjected to at least three freeze-thaw cycles, and further disrupted by ultrasonication. After centrifugation, the supernatant was collected, mixed with 5 mL of 1%-2% (v/v) nitric acid, and analyzed by inductively coupled plasma (ICP) spectrometry to quantify intracellular copper content. This experiment was designed to determine how the binding strategy affected the diffusion efficiency of H-Cu_2_O nanozymes into inflamed cells.

***In Vivo* Characterisation and Multi-Omics Analysis**

**Disease activity index (DAI) and organ indices:** Body weight, stool consistency, and gross rectal bleeding were monitored daily. DAI was calculated according to the following established criteria: Score 0, no weight loss, normal well-formed stool, and occult blood negative; Score 1, 1-5% weight loss, normal stool, and no bleeding; Score 2, 6-10% weight loss, loose stools, and occult blood positive; Score 3, 11-18% weight loss, loose stools with gross bleeding; and Score 4, >18% weight loss, diarrhea, and gross bleeding. At sacrifice, the liver and spleen were excised and weighed. Organ indices were calculated as follows: liver index (%) = [liver weight (g) / body weight (g)] × 100; spleen index (mg g^-1^) = spleen weight (mg) / body weight (g).

**Histopathology and immunohistochemistry (IHC):** Excised colon tissues were fixed, paraffin-embedded, and sectioned at 4 µm. Sections were stained with hematoxylin and eosin (H&E) for structural evaluation. Histological scoring on a 0-4 scale comprehensively assessed inflammation severity, mucosal/crypt damage, and lesion extent. Alcian Blue-Periodic Acid-Schiff (AB-PAS) staining was performed according to the manufacturer’s instructions to assess goblet-cell populations, followed by quantitative analysis using ImageJ. For IHC, deparaffinized sections underwent antigen retrieval in EDTA buffer. Endogenous peroxidase activity was quenched with 3% H_2_O_2_, followed by serum blocking. Sections were incubated overnight at 4 °C with specific primary antibodies and then with the corresponding secondary antibodies. Signals were developed using 3,3′-diaminobenzidine (DAB), and sections were counterstained with hematoxylin. Images were acquired by light microscopy and quantified using ImageJ.

**RNA extraction and qRT-PCR:** Total RNA was isolated from colon tissues and cultured cells using TRIzol reagent. After quantification, RNA was reverse-transcribed into cDNA. Quantitative real-time PCR was performed using a SYBR Green master-mix system. β-Actin or GAPDH served as the endogenous reference genes. Relative mRNA expression levels were calculated using the 2^−ΔΔCt method. Primer sequences are listed in **Table S1** (**Supporting Information**).

**Western blot:** Protein samples were separated by SDS-PAGE and transferred onto pre-activated PVDF membranes. After blocking with 5% non-fat milk, membranes were incubated with primary antibodies overnight at 4 °C, followed by incubation with the corresponding secondary antibodies. Protein bands were visualized using enhanced chemiluminescence, and band intensities were quantified using ImageJ. Relative protein expression was normalized to GAPDH.

**Fecal microbiota profiling (16S rRNA gene sequencing):** Fresh fecal pellets (≥3 per mouse) were collected under sterile conditions and stored at low temperatures. Microbial genomic DNA extraction, 16S rRNA gene amplicon sequencing, and comprehensive bioinformatic analyses, including β-diversity, principal coordinate analysis (PCoA), and differential taxonomic abundance analysis, were performed using the Majorbio Cloud Platform.

**Biodistribution and tissue copper quantification:** To assess potential systemic metal accumulation, liver and kidney tissues were randomly sampled from the Control, H-Cu_2_O, and combinatorial treatment groups (n = 3/group). Tissues were accurately weighed, homogenized, and centrifuged. The resulting supernatants were filtered through a 40 µm membrane before elemental copper quantification by inductively coupled plasma (ICP) spectrometry.

**Hemolysis rate:** Whole blood was collected from the C57BL/6J mice via the orbital venous plexus and centrifuged to isolate red blood cells. Erythrocytes were washed repeatedly with PBS until the supernatant became clear and then diluted with PBS to obtain a 4% (v/v) erythrocyte suspension. After centrifugation, the erythrocyte pellet was resuspended in PBS containing different concentrations of test samples. Normal saline and distilled water were used as the negative and positive controls, respectively. After incubation at 37 °C for 3 h, the mixtures were centrifuged, and absorbance of the supernatant was measured at 577 nm. Hemolysis rate was calculated as follows: Hemolysis rate (%)=[(A_sample_ - A_negative control_)/(A_positive control_ - A_negative control_)] × 100%.

**Statistical analysis**

Data are presented as mean ± standard deviation (SD). Statistical analyses were performed using GraphPad Prism (version 8.0). Two-group comparisons were conducted using Student’s t-test, and multiple-group comparisons were analyzed by one-way ANOVA with appropriate multiple-comparison correction. A *P* value < 0.05 was considered statistically significant. Significance was denoted as ^#^*P*<0.05, ^##^*P*<0.01, ^###^*P*<0.001 versus the control group, and ^*^*P*<0.05, ^**^*P*<0.01, ^***^*P*<0.001 versus the model (DSS) group.

**
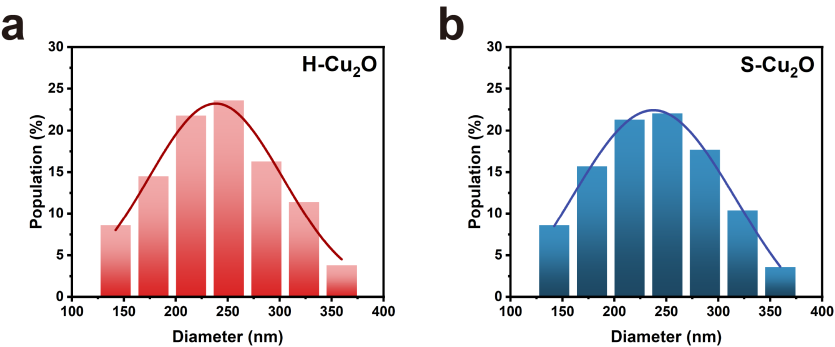
**

**Figure S1.** Particle size distributions of H-Cu_2_O (a) and S-Cu_2_O (b) nanozymes.

**Figure S2.** Zeta potentials of H-Cu_2_O and S-Cu_2_O nanozymes.


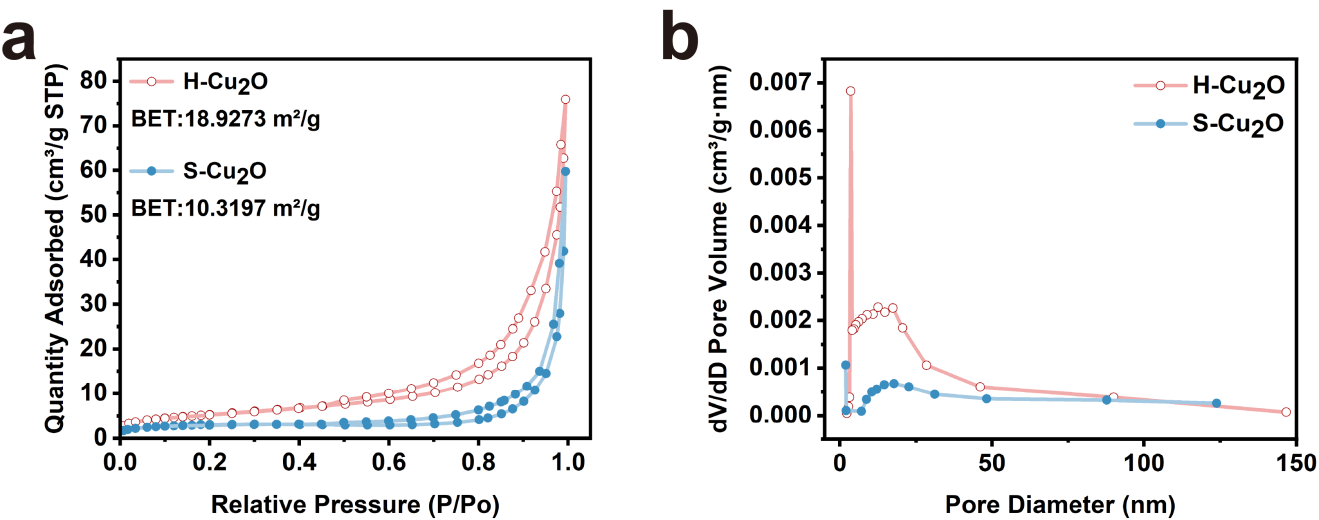


**Figure S3.** (a) N_2_ adsorption-desorption isotherms and (b) pore-size distribution curves of H-Cu_2_O and S-Cu_2_O nanozymes.

**
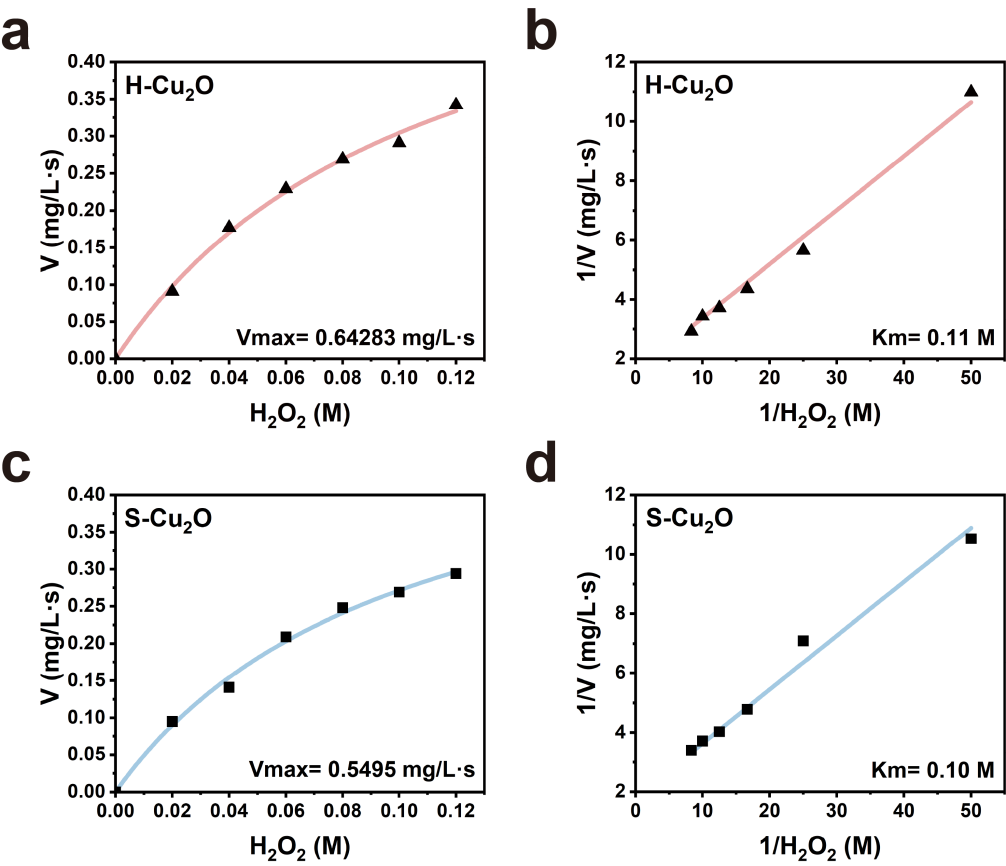
**

**Figure S4.** Michaelis–Menten kinetic curves and Lineweaver–Burk plots of the catalase-like activity of H-Cu_2_O and S-Cu_2_O nanozymes. (a, b) Kinetic analysis of H-Cu_2_O. (c, d) Kinetic analysis of S-Cu_2_O.

**
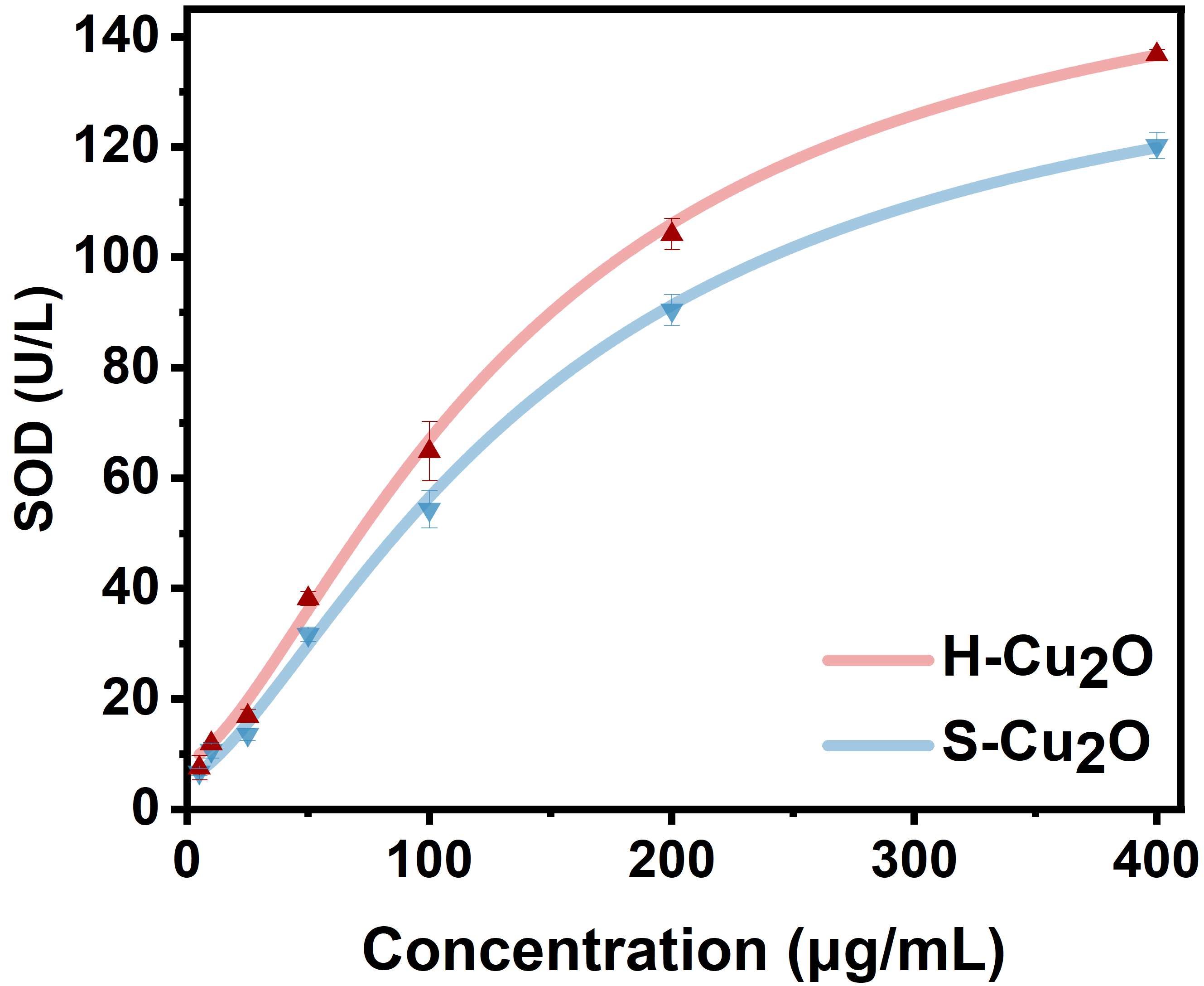
**

**Figure S5.** SOD-like activity of H-Cu_2_O and S-Cu_2_O nanozymes.

**
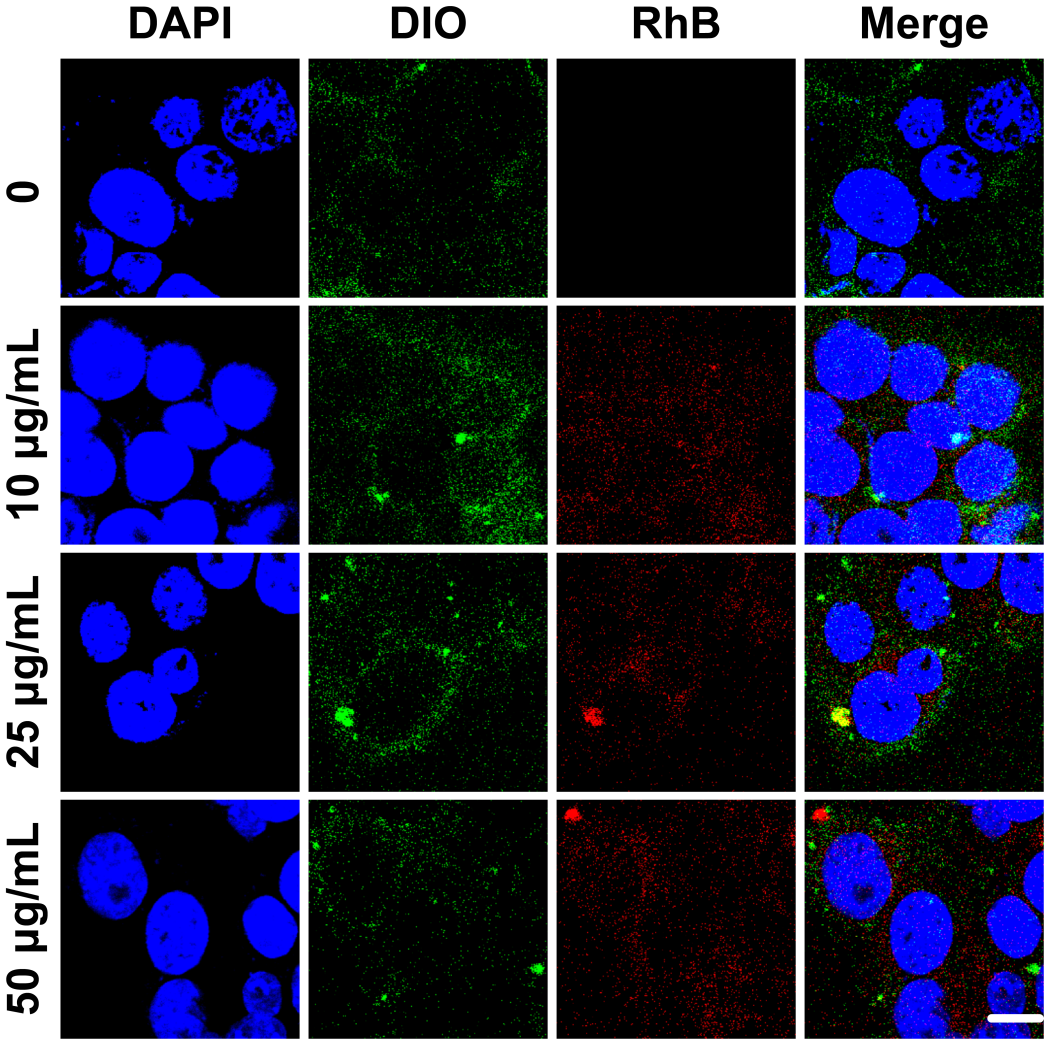
**

**Figure S6.** Concentration-dependent cellular uptake of H-Cu_2_O in NCM460 cells (scale bar: 10 μm).

**
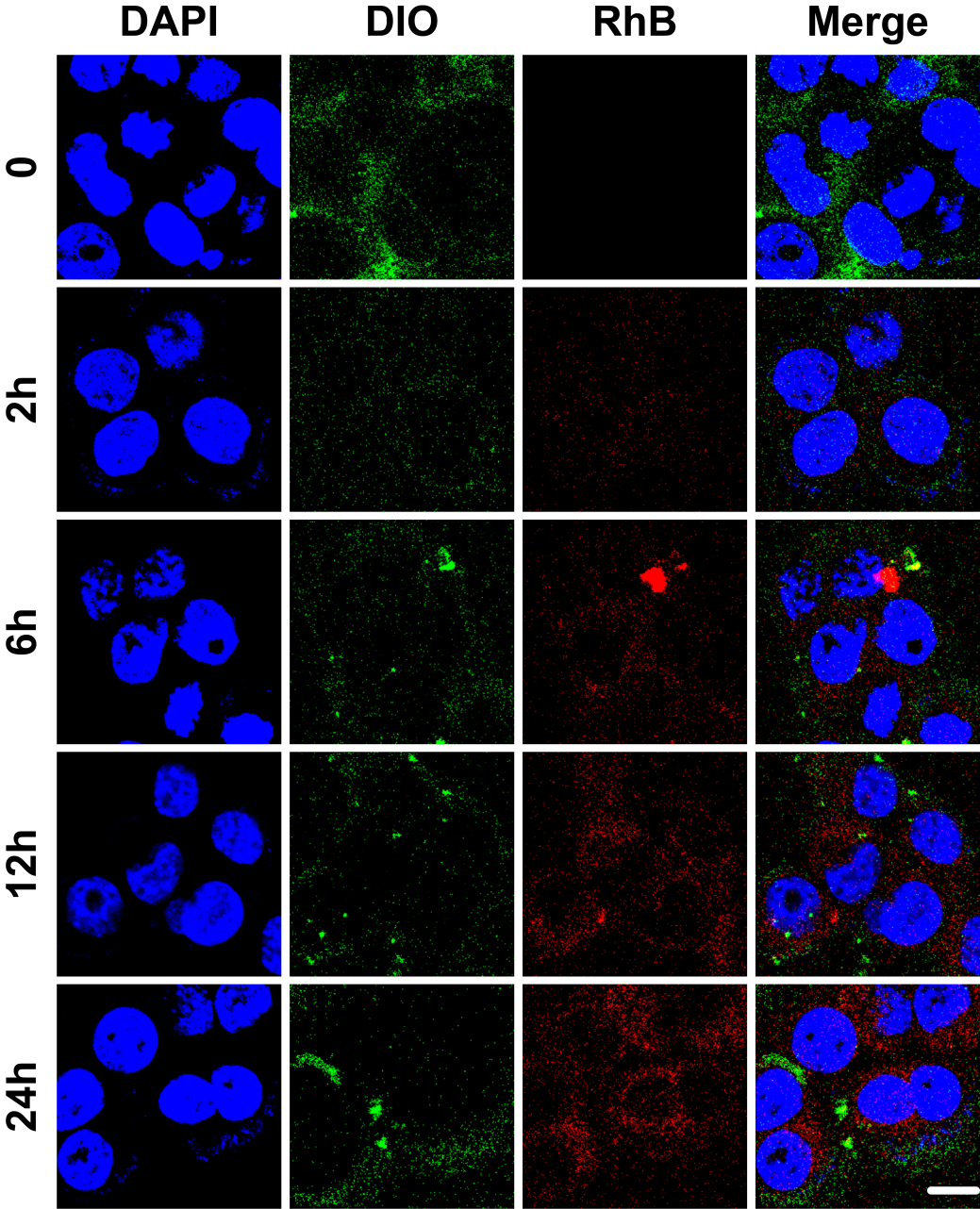
**

**Figure S7.** Time-dependent cellular uptake of H-Cu_2_O in NCM460 cells (scale bar: 10 μm).

**
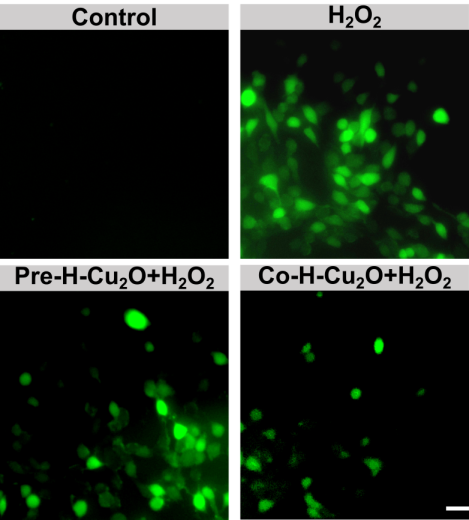
**

**Figure S8.** Intracellular ROS levels in H_2_O_2_-challenged NCM-460 cells after H-Cu_2_O intervention (50 µg mL^-1^), as visualized by DCFH-DA fluorescence imaging (scale bar: 30 μm).

**Figure S9.** Changes in the disease activity index (DAI) in mice during the H-Cu_2_O treatment period.

**
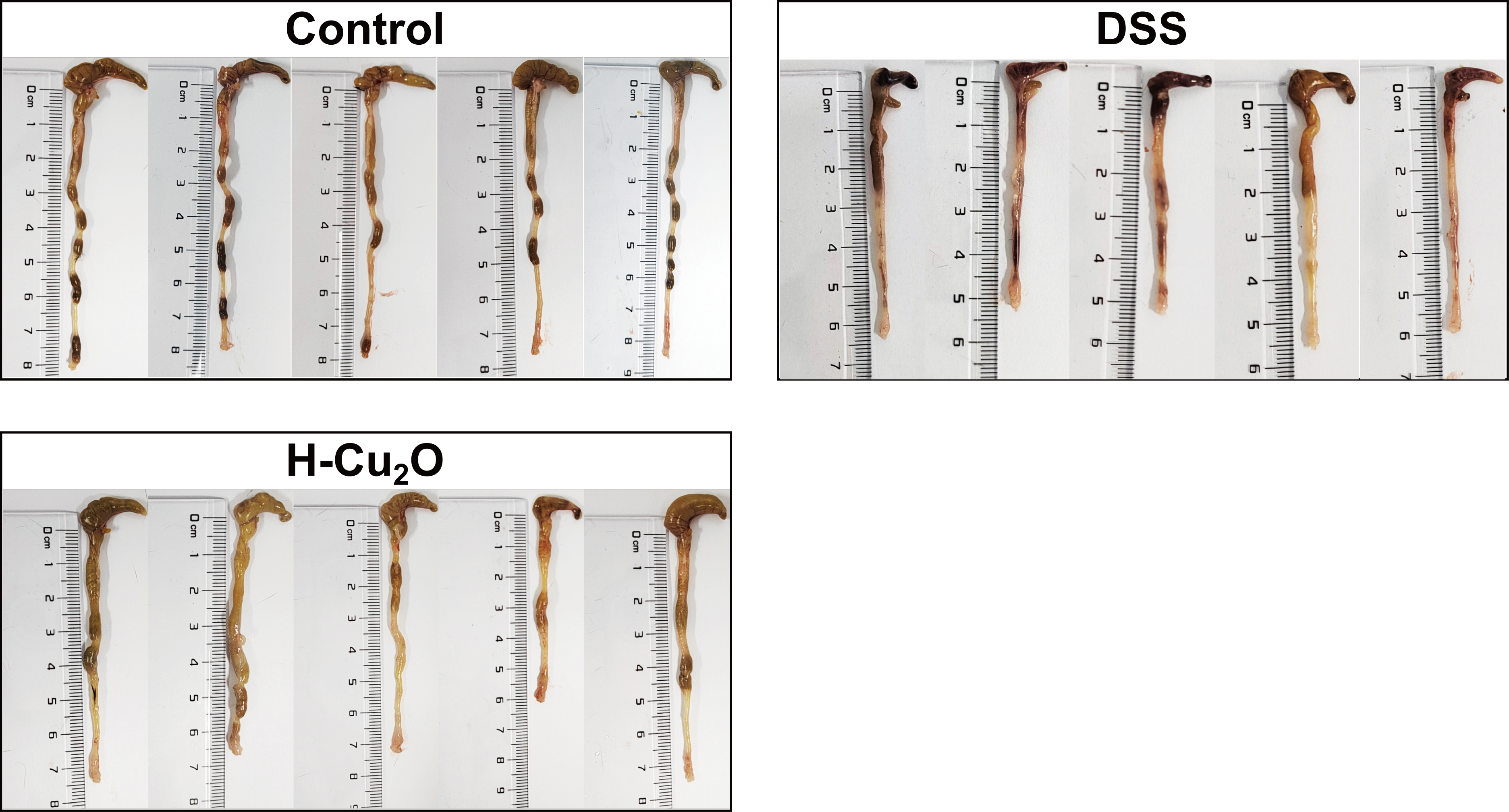
**

**Figure S10.** Representative macroscopic images of colons (n=6).

**Figure S11.** Quantitative assessment of colonic histopathological scores. Data are presented as mean ± SD. ^#^*P* < 0.05, ^##^*P* < 0.01, ^###^*P* < 0.001 (*vs*. Control); ^*^*P* < 0.05, ^**^*P* < 0.01, ^***^*P* < 0.001 (*vs*. DSS).

**Figure S12.** Quantification of goblet cell numbers in colonic sections based on PAS staining. Data are presented as mean ± SD. ^#^*P* < 0.05, ^##^*P* < 0.01, ^###^*P* < 0.001 (*vs*. Control); ^*^*P* < 0.05, ^**^*P* < 0.01, ^***^*P* < 0.001 (*vs*. DSS).

**Figure S13.** Quantitative analysis of the mean optical density (MOD) for ZO-1 immunostaining. Data are presented as mean ± SD. ^#^*P* < 0.05, ^##^*P* < 0.01, ^###^*P* < 0.001 (*vs*. Control); ^*^*P* < 0.05, ^**^*P* < 0.01, ^***^*P* < 0.001 (*vs*. DSS).

**Figure S14.** Quantitative analysis of the mean optical density (MOD) for Occludin immunostaining. Data are presented as mean ± SD. ^#^*P* < 0.05, ^##^*P* < 0.01, ^###^*P* < 0.001 (*vs*. Control); ^*^*P* < 0.05, ^**^*P* < 0.01, ^***^*P* < 0.001 (*vs*. DSS).

**
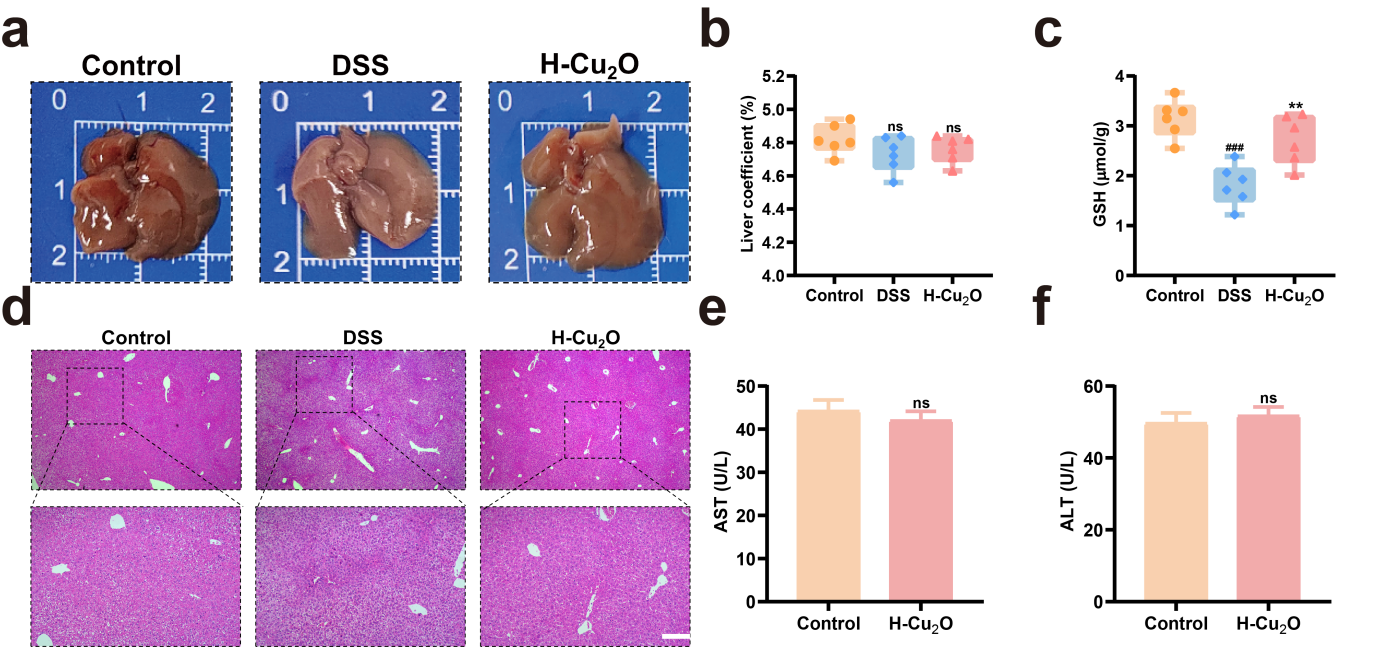
**

**Figure S15.** *In vivo* safety evaluation of H-Cu_2_O nanozymes in DSS-induced colitis mice. (a) Representative gross morphological images of livers. (b) Liver index. (c) Glutathione (GSH) levels in serum. (d) Representative H&E stained liver sections, with magnified views provided below (scale bar: 100 μm). (e, f) Aspartate aminotransferase (AST, e) and alanine aminotransferase (ALT, f) in serum. Data are presented as mean ± SD. ^#^*P* < 0.05, ^##^*P* < 0.01, ^###^*P* < 0.001 (*vs*. Control); ^*^*P* < 0.05, ^**^*P* < 0.01, ^***^*P* < 0.001 (*vs*. DSS); ns: not significant .

**Figure S16.** Relative mRNA expression levels of *NLRP3* in **RAW264.7 cells***.* Data are presented as mean ± SD. ^#^*P* < 0.05, ^##^*P* < 0.01, ^###^*P* < 0.001 (*vs*. Control); ^*^*P* < 0.05, ^**^*P* < 0.01, ^***^*P* < 0.001 (*vs*. DSS).

**Figure S17.** Relative mRNA expression levels of *IL-1β* in **RAW264.7 cells.** Data are presented as mean ± SD. ^#^*P* < 0.05, ^##^*P* < 0.01, ^###^*P* < 0.001 (*vs*. Control); ^*^*P* < 0.05, ^**^*P* < 0.01, ^***^*P* < 0.001 (*vs*. DSS); ns: not significant (*vs*. DSS).


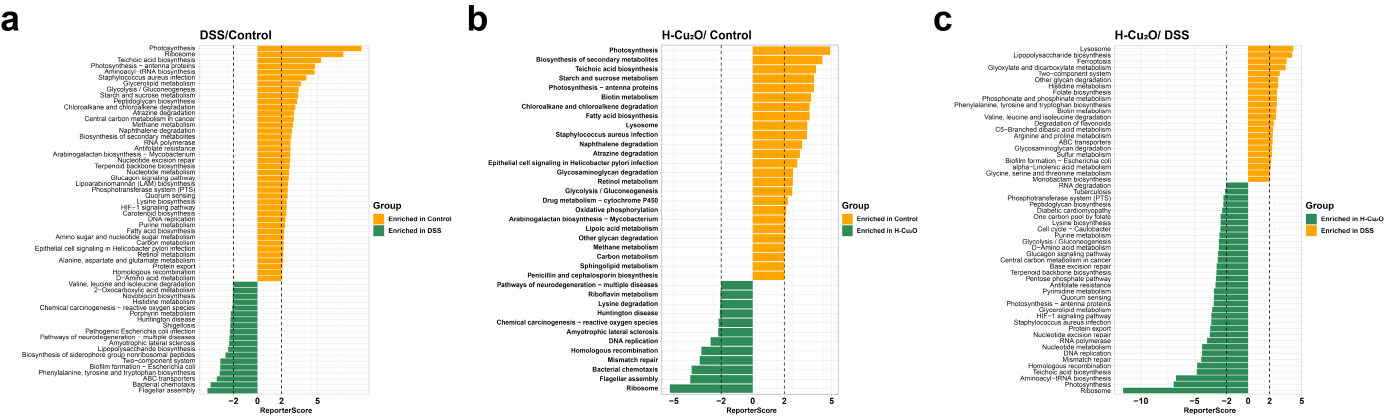


**Figure S18.** Differential functional pathway analysis of the gut microbiota predicted from 16S rRNA gene sequences.

**
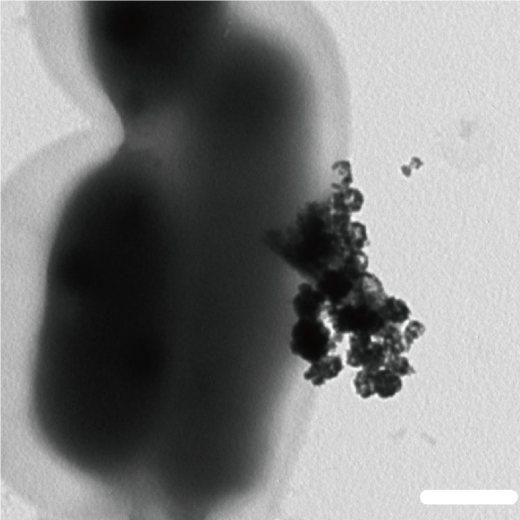
**

**Figure S19.** Representative transmission electron microscopy image of the electrostatic complex formed between *Lactobacillus plantarum* and H-Cu_2_O nanozymes (LP@H-Cu_2_O) (scale bar: 500 nm).

**Figure S20.** Zeta potentials of LP, H-Cu_2_O, and LP@H-Cu_2_O.

**Figure S21.** Growth kinetics (OD_600 nm_) of *L. plantarum* under different intervention conditions (n = 3).

**Figure S22.** Copper ion content in NCM460 cells in each group. Transwell-ICP quantification of H-Cu_2_O delivered to lower compartment cells from LP + H-Cu_2_O *vs* LP@H-Cu_2_O at 25 and 50 µg mL^-1^.

**Figure S23.** Relative mRNA expression levels of *IL-1β* in Transwell experiment*.* Data are presented as mean ± SD. ^#^*P* < 0.05, ^##^*P* < 0.01, ^###^*P* < 0.001 (*vs*. Control); ^*^*P* < 0.05, ^**^*P* < 0.01, ^***^*P* < 0.001 (*vs*. DSS).

**
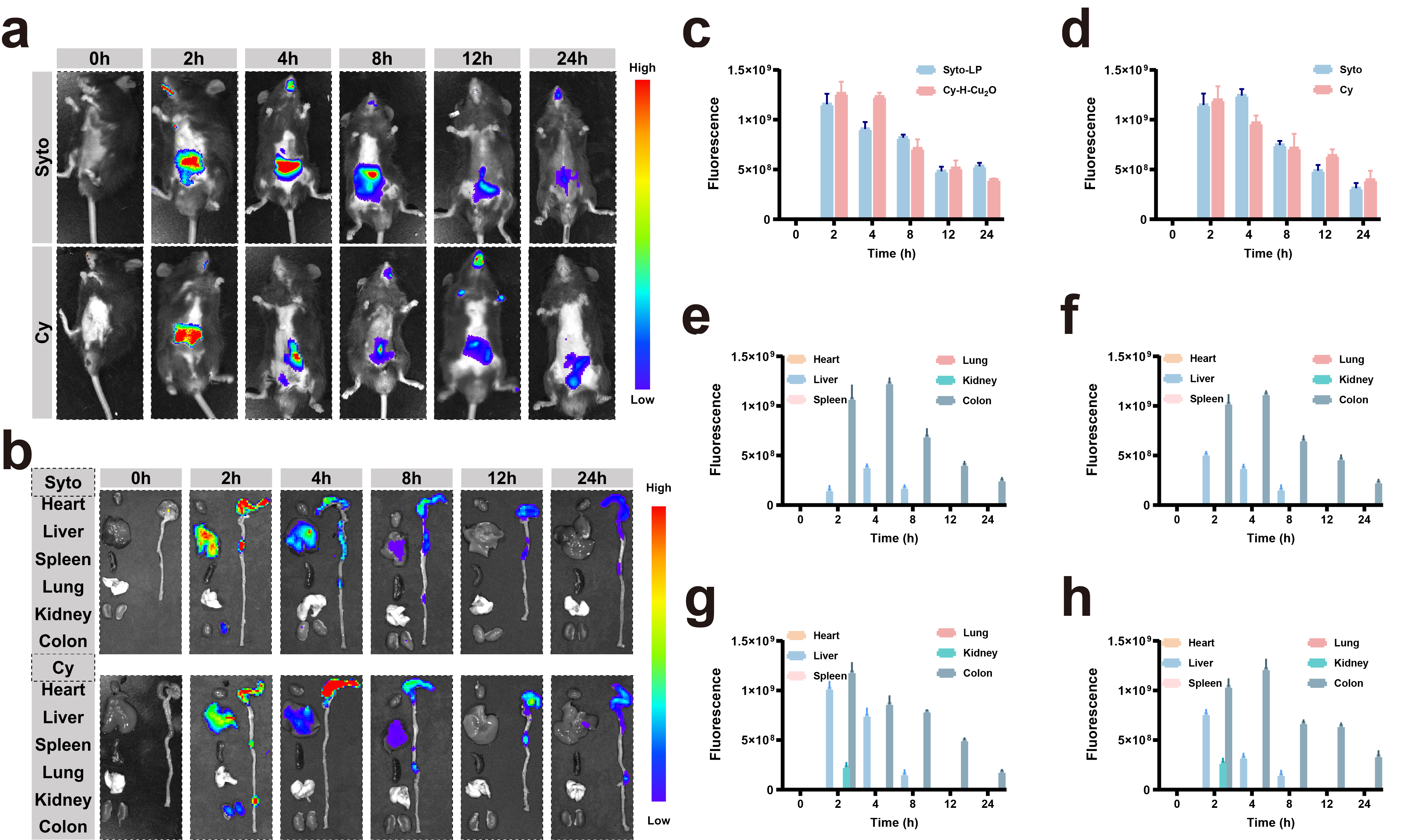
**

**Figure S24. *In vivo* and ex vivo fluorescence imaging of bacteria and nanozymes.** (a) *In vivo* fluorescence images showing the biodistribution of orally administered free Syto dye and free Cy dye from 0 to 24 h. (b) Ex vivo fluorescence images of major organs and colon tissues at the indicated time points. (c, d) Quantification of *in vivo* fluorescence intensity for Syto-labeled *Lactobacillus plantarum* and Cy-labeled H-Cu_2_O (c), and free Syto and Cy dyes (d) (n = 3). (e-h) Quantification of ex vivo fluorescence intensity in major organs and intestinal tissues from the Syto-LP (e), Cy-H-Cu_2_O (f), free Syto dye (g), and free Cy dye (h) groups (n = 3). Data are presented as mean ± SD.

**Figure S25.** Changes in the disease activity index (DAI) of mice during the combinatorial treatment period.

**
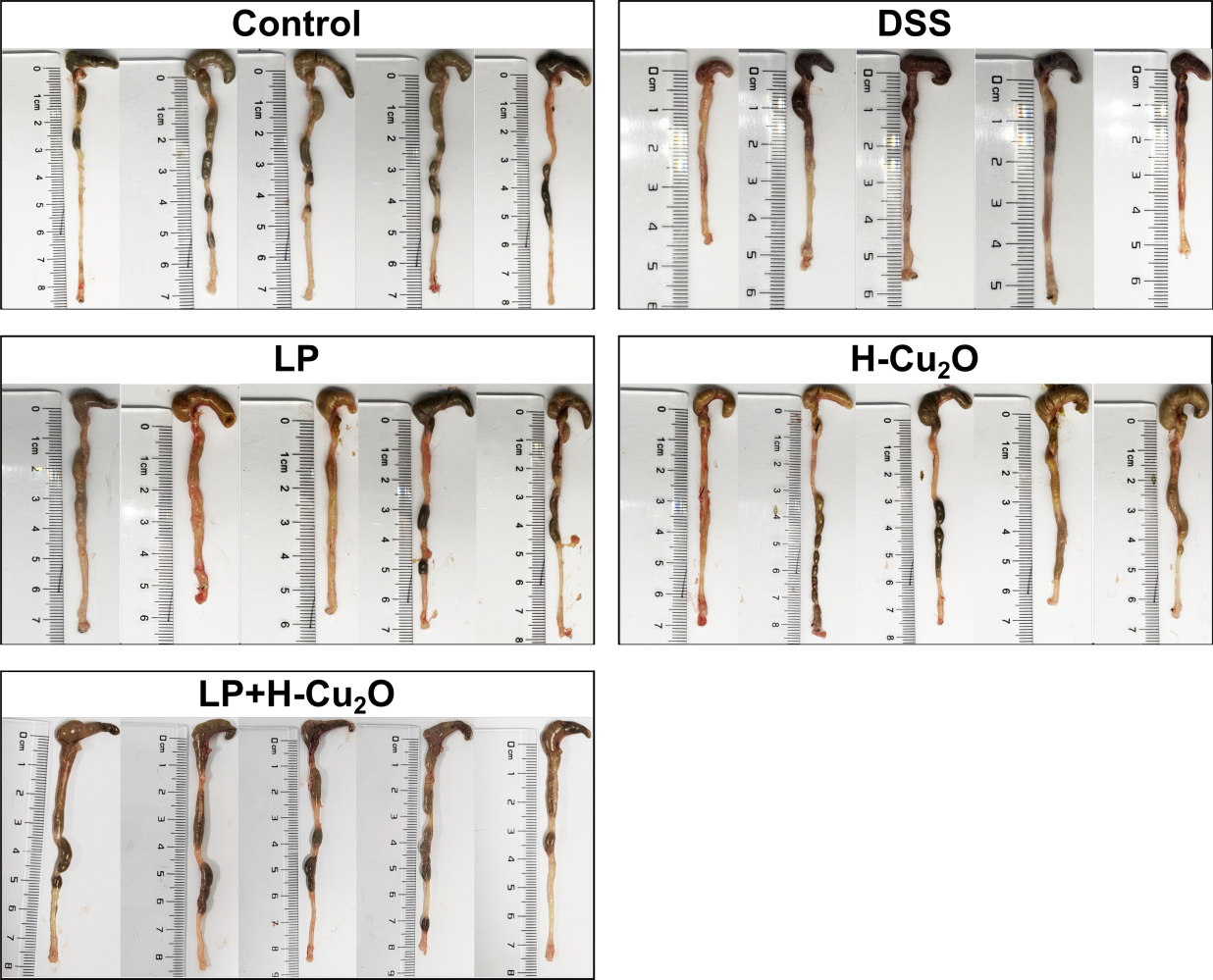
**

**Figure S26.** Representative macroscopic images of colons (n=6).

**Figure S27.** Relative colonic mRNA expression levels of *Occludin.* Data are presented as mean ± SD. ^#^*P* < 0.05, ^##^*P* < 0.01, ^###^*P* < 0.001 (*vs*. Control); ^*^*P* < 0.05, ^**^*P* < 0.01, ^***^*P* < 0.001 (*vs*. DSS).

**Figure S28.** Relative colonic mRNA expression levels of *Claudin-1.* Data are presented as mean ± SD. ^#^*P* < 0.05, ^##^*P* < 0.01, ^###^*P* < 0.001 (*vs*. Control); ^*^*P* < 0.05, ^**^*P* < 0.01, ^***^*P* < 0.001 (*vs*. DSS).

**
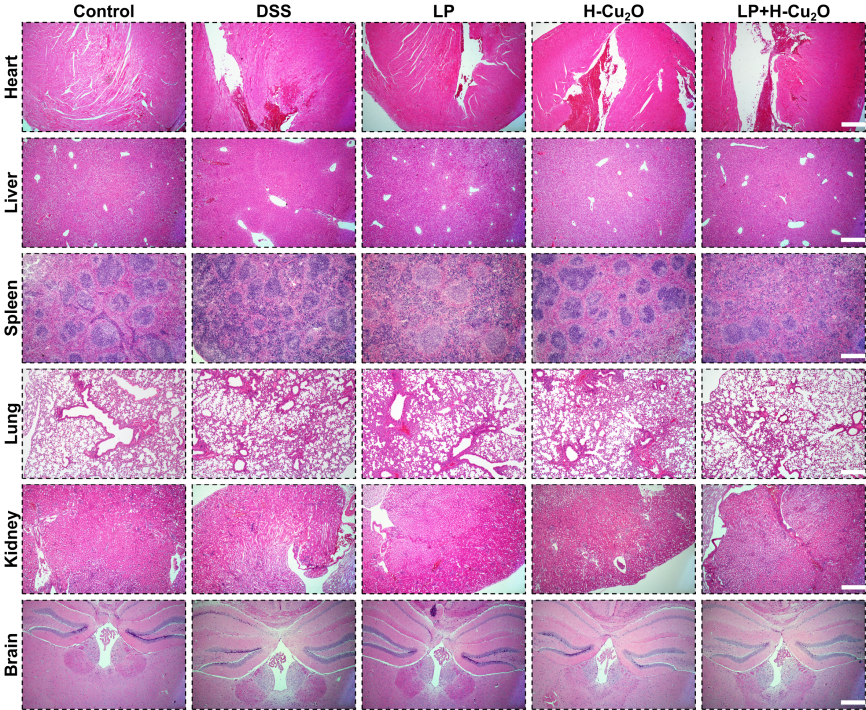
**

**Figure S29.** Representative H&E-stained sections of major organs (heart, liver, spleen, lung, kidney, and brain; scale bars: 50 μm) . No significant systemic histological toxicity was observed.

**Figure S30.** Copper ion content in the liver in each group. Data are presented as mean ± SD. ^#^*P* < 0.05, ^##^*P* < 0.01, ^###^*P* < 0.001 (*vs*. Control); ns: not significant (*vs*. Control).

**Figure S31.** Copper ion content in the kidney in each group. Data are presented as mean ± SD. ns: not significant (*vs*. Control).

**
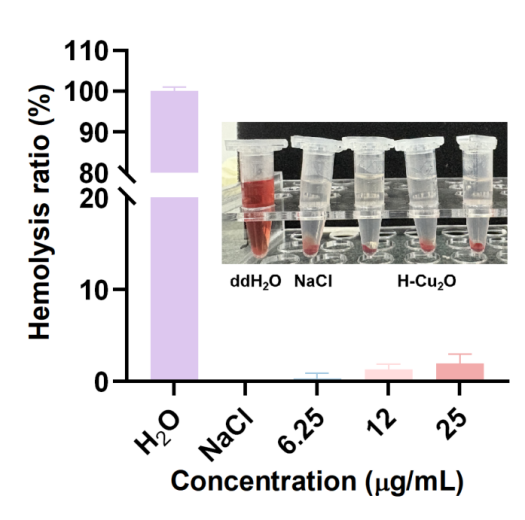
**

**Figure S32.** Hemolysis assay evaluating the blood biocompatibility of H-Cu_2_O nanozymes (n=3).

**GAPDH:**

**
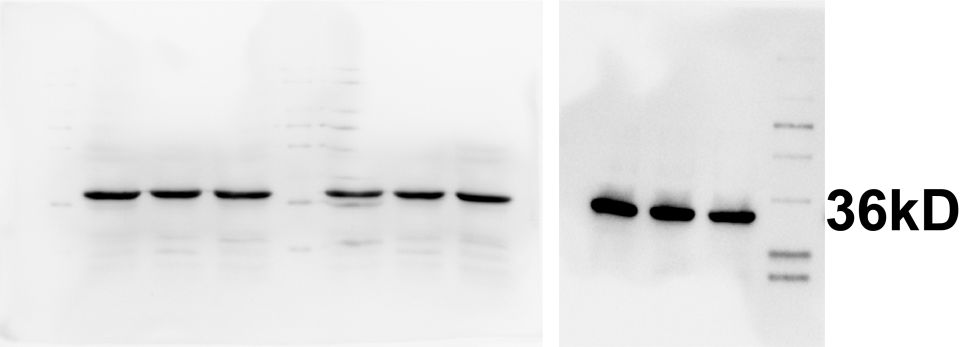
**

**Figure S33.** Uncropped Western blot images corresponding to Figure 5d (Loading control: GAPDH, ~36 kDa).

**TXNIP:**

**
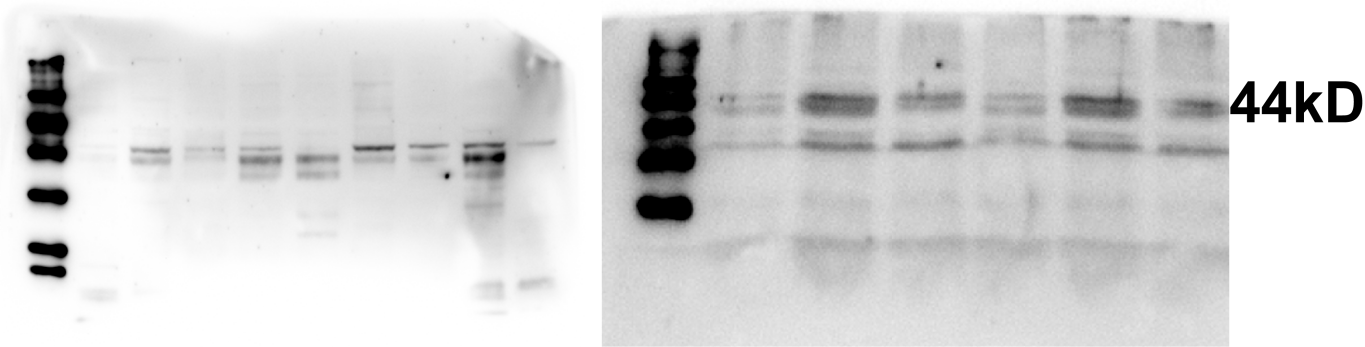
**

**Figure S34.** Uncropped Western blot images corresponding to Figure 5d. (TXNIP, ~44kDa).

**Caspase-1:**

**
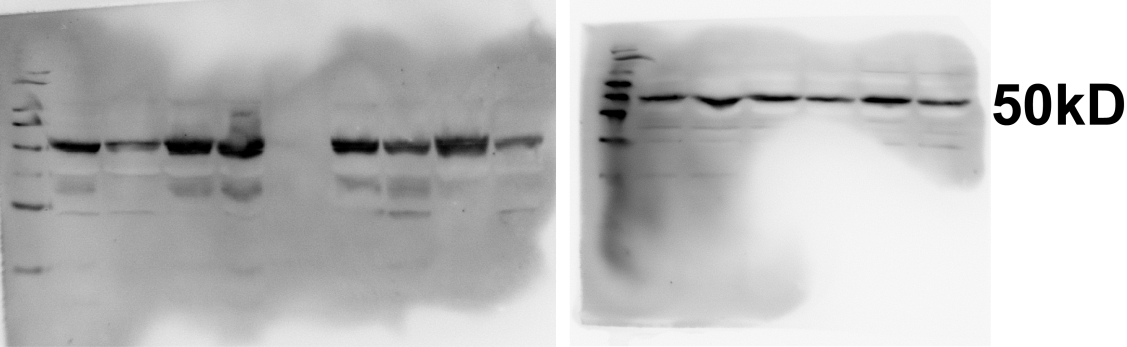
**

**Figure S35.** Uncropped Western blot images corresponding to Figure 5h (Pro-Caspase-1, ~50 kDa).

**Cleaved caspase-1:**

**
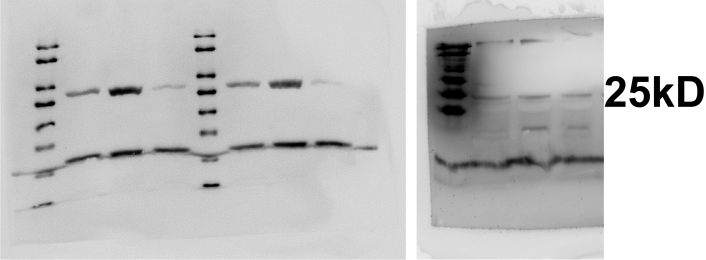
**

**Figure S36.** Uncropped Western blot images corresponding to Figure 5h (Cleaved Caspase-1, ~25 kDa).

**IL-1β:**

**
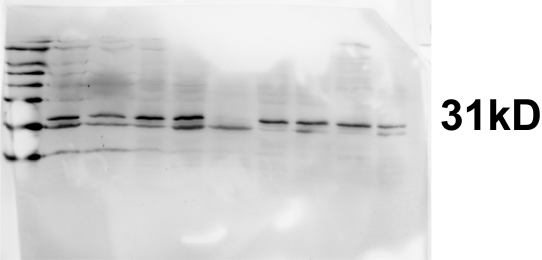
**

**Figure S37.** Uncropped Western blot images corresponding to Figure 5l (Pro-IL-1β, ~31 kDa).

**Cleaved IL-1β:**

**
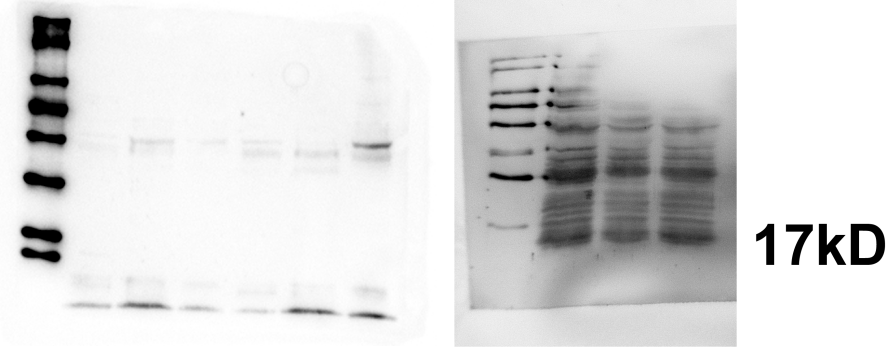
**

**Figure S38.** Uncropped Western blot images corresponding to Figure 5l (Cleaved IL-1β, ~17 kDa).

**Table S1.** The primer sequences used in this study.

| Gene | Primer sequence |
| --- | --- |
| *β-actin* | F: CATGTACGTTGCTATCCAGGC  R: CTCCTTAATGTCACGCACGAT |
| *Gapdh* | F: GGAAGGGCTCATGACCACAG  R: TCACGCCACAGCTTTCCAG |
| *ZO-1* | F: TCTTCCATCATTTCGCTGTGT  R: TCTGAAACCATCAAGTCCACA |
| *Occludin* | F: TCACTTTTCCTGCGGTGACT  R: GGGAACGTGGCCGATATAATG |
| *Claudin-1* | F:ATGCAAAGATGTTTTGCCACAG  R: TACAAATTCCCATTGCAGCCC |
| *Muc2* | F: AGGCTCGGAACTCCAGAAA  R: CCAGGGAATCGGTAGACATCG |
| *Nrf2* | F: TCTTGGAGTAAGTCGAGAAGTGT  R: GTTGAAACTGAGCGAAAAAGGC |
| *Trx-1* | F: TCTGTCACGGATTCCTCAT  R: GTTTCCCAGTGTACTGCTTT |
| *TXNIP* | F: TGTGTGAAGTTACTCGTGTCAAA  R: GCAGGTACTCCGAAGTCTGT |
| *NLRP3* | F: TGGAAGATTACCCGCCCGAGAA  R: TCCCAGCAAACCCATCCACTCTTC |
| *ASC* | F: CATCTTGTCTTGGCTGGTGGTCT  R: CGGACACGGACAGGATTGACA |
| *Caspase-1* | F: TGAAAGACAAGCCCAAGGTGATC  R: CAATGAAAAGTGAGCCCCTGAC |
| *TNF-ɑ* | F: ATGAGAAGTTCCCAAATGGC  R: CTCCACTTGGTGGTTTGCTA |
| *IL-6* | F: CCCTTTGCTATGGTGTCCTT  R: TGGTTTCTCTTCCCAAGACC |
| *IL-1β* | F: TGCCACCTTTTGACAGTGATG  R: AAGGTCCACGGGAAAGACAC |
| *IL-18* | F: TCAGACAACTTTGGCCGACT  R: TCAGTCTGGTCTGGGGTTCA |
| *A.muciniphila* | F: CAGCACGTGAAGGTGGGGAC  R: CCTTGCGGTTGGCTTCAGAT |
| *L. plantarum* | F: TGACAACCATTCCATCACGTT  R: CCTTACGCTCACCATCACCT |
| *L.acidophilus* | F: GAAAGAGCCCAAACCAAGTGATT  R: CTTCCCAGATAATTCAACTATCGATTA |
